# Viewed touch influences tactile detection by altering decision criterion

**DOI:** 10.3758/s13414-024-02959-7

**Published:** 2024-11-05

**Authors:** Anupama Nair, Jared Medina

**Affiliations:** 1 Department of Psychological and Brain Sciences, University of Delaware, 105 The Green, Room 108, Newark, DE 19716 USA; 2 The Department of Psychology, Emory University, 36 Eagle Row, Atlanta, GA 30322 USA

**Keywords:** Vicarious tactile activation, Signal detection theory, Tactile perception, Tactile sensitivity, Decision criterion

## Abstract

Our tactile perception is shaped not only by somatosensory input but also by visual information. Prior research on the effect of viewing touch on tactile processing has found higher tactile detection rates when paired with viewed touch versus a control visual stimulus. Therefore, some have proposed a vicarious tactile system that activates somatosensory areas when viewing touch, resulting in enhanced tactile perception. However, we propose an alternative explanation: Viewing touch makes the observer more liberal in their decision to report a tactile stimulus relative to not viewing touch, also resulting in higher tactile detection rates. To disambiguate between the two explanations, we examined the effect of viewed touch on tactile sensitivity and decision criterion using signal detection theory. In three experiments, participants engaged in a tactile detection task while viewing a hand being touched or approached by a finger, a red dot, or no stimulus. We found that viewing touch led to a consistent, liberal criterion shift but inconsistent enhancement in tactile sensitivity relative to not viewing touch. Moreover, observing a finger approach the hand was sufficient to bias the criterion. These findings suggest that viewing touch influences tactile performance by altering tactile decision mechanisms rather than the tactile perceptual signal.

**Supplementary Information:**

The online version contains supplementary material available at 10.3758/s13414-024-02959-7.

## Introduction

Perception of a tactile stimulus can be determined by both inputs from the somatosensory system and inputs in other modalities that occur at the same time and location as the tactile stimulus. For example, vision of the body has been shown to improve tactile detection (Kennett et al., 2001; Press et al., 2004; Taylor-Clarke et al., 2002). More specifically, presenting a visual stimulus synchronously with a tactile stimulus has also been shown to change performance on tactile detection tasks. For example, Serino et al. (2008) examined participants’ performance when they viewed videos of a person’s face being touched or approached by fingers on one or both sides while being presented with concurrent tactile stimulation (electrical stimuli) on one or both of their own cheeks. Here, the visual stimulus was a video of touch presented to another person’s face. Participants were instructed to detect tactile stimulation on their own face while watching the videos. The authors hypothesized that if viewing touch affected tactile perception, then the effect of viewing touch on tactile behavioral performance should be greater than the control approach stimulus. The bilateral tactile stimulation condition (i.e., electrical stimuli on both cheeks) was associated with a higher percentage of correct responses when viewing videos of touch versus approach (see also Rorden et al., 1999). Consistent with this hypothesis, viewing touch on the body activates areas in primary (SI) and/or secondary (SII) somatosensory cortex that are also active when individuals are actually touched (Blakemore et al., 2005; Cardini et al., 2011; Ebisch et al., 2008, 2011; Holle et al., 2013; Keysers et al., 2004; Mccabe et al., 2008; Meyer et al., 2011; Pihko et al., 2010; Schaefer et al., 2009a, 2009b, 2012, 2013; Streltsova & McCleery, 2014). Such evidence has given rise to the theory that there exists a shared neural circuitry for touch (“mirror system for touch”) that responds to both tactile stimulation of one’s body and visual stimulation depicting touch on someone else (Blakemore et al., 2005; Ebisch et al., 2008; Keysers et al., 2004; Pihko et al., 2010; Schaefer et al., 2009a, 2009b). Such activation for observed touch (or vicarious activation) is thought to represent modulation of tactile perceptual activity (Blakemore et al., 2005; Cardini et al., 2011; Ebisch et al., 2008).

However, an alternative interpretation is that prior results are due to changes in decision thresholds to report a tactile stimulus when a visual touch stimulus is present. Consider an observer who is more liberal in judging what constitutes a tactile signal when a visual touch stimulus is present. In a tactile detection task, such an observer is more likely to respond that a tactile stimulus is present when visual touch is presented, irrespective of whether they perceive the tactile stimulus. Such a liberal bias will also lead to higher detection of the tactile stimulus because there is a greater probability of correctly responding to the tactile stimulus when it is present. However, this increase in detection rate would not reflect enhanced tactile perception.

In studies that have supported the perceptual enhancement account, researchers have typically examined the effect of viewed touch by comparing trials where visual touch is present versus absent, but the tactile stimulus is always present. In such a design, any increase in accuracy with visual touch (versus the control condition) could be due to either an enhancement in tactile signal or a liberal response bias due to the presence of viewed touch. To disambiguate between the two possibilities, one would need a measure of tactile sensitivity (i.e., ability to detect signal in the stimulus (McNicol, 2004)) and decision criterion (i.e., a decision threshold based on whether a trial is classified as signal or noise (McNicol, 2004)) to determine the specific effect of visual touch on tactile performance. Signal detection measures d’ and c provide indices of sensitivity and decision criterion, respectively (Green & Swets, 1966; Swets et al., 1961) based not only on trials where the signal of interest is present but also when it is absent. A higher d’ score relative to a comparison condition reflects increased sensitivity to the signal, which, in turn, indicates better perception. A lower c score relative to a comparison condition reflects a decrease in decision criterion, which suggests a greater liberal bias to respond to the stimulus (McNicol, 2004; Stanislaw & Todorov, 1999).

Signal detection measures have been used to examine the effect of vision on tactile processing, where the visual stimulus is in the same location as the tactile stimulus. In the Somatic Signal Detection Task (SSDT), participants are presented with near-threshold tactile stimuli on their finger in the presence or absence of visual stimuli (in this case, a red LED flash presented close to the stimulated finger) that is non-predictive, i.e., does not always occur with a tactile stimulus (Durlik et al., 2014; Johnson et al., 2006; Lloyd et al., 2008; McKenzie et al., 2010; Mirams et al., 2010, 2012, 2017). The authors hypothesized that if the presence of the visual stimulus enhanced tactile sensitivity, it should lead to an increase in d’ compared to its absence. However, the results for d’ were mixed and inconsistent. For example, Johnson et al. (2006) did not find the visual stimulus to significantly increase d’ relative to its absence in any of their within-subjects comparison, even though the visual stimulus significantly improved tactile detection rates. They found a significant effect of the visual stimulus on tactile sensitivity (i.e., d’ was higher when the visual stimulus was present versus absent) only when they conducted a between-subjects comparison to determine whether there were any overall differences between their experiments. In one study by McKenzie et al. (2010), the visual light flash enhanced tactile sensitivity relative to the absence of the flash only when the trial start cue (indicating start of trial) was visual or auditory, but not when it was tactile. In another study, the light flash did not enhance tactile sensitivity compared to no light flash (Mirams et al., 2012). Thus, the effects of vision on tactile sensitivity are not consistent and are often modulated by other factors in the experiment.

Contrasting the results of sensitivity with that of criterion, a liberal criterion shift with the visual stimulus was consistently present. For example, in the study by Johnson et al. (2006) discussed earlier, participants’ criterion was significantly more liberal when the visual stimulus was present versus absent, both in their within- and between-experiment comparisons. Evidence for a liberal shift in criterion in the presence versus absence of the visual stimulus has also been consistently reported in other studies that have adopted the SSDT (Durlik et al., 2014; McKenzie et al., 2010; Mirams et al., 2012, 2017) and in some studies that have examined visual remapping of touch (Cardini et al., 2013).

While findings from the SSDT studies are noteworthy and highlight the robust effect of light flashes on tactile decision criterion, they cannot generalize to the findings of the visual touch literature. Unlike the SSDT studies where the authors observe an effect of vision on tactile performance when it occurs in the same location as the tactile stimulus, the effect of visual touch is observed even when it occurs in a different location from the tactile stimulus on one’s body. It is hypothesized that the effects of visual touch are driven by vicarious activation of the tactile perceptual system, which may go beyond simple visual-tactile integration effects from seeing a concurrent light flash in the same location as touch. Given the possibility that distinct mechanisms may underlie the influence of visual touch and light flash on tactile performance, their effects should be examined separately. The possibility of distinct mechanisms would also suggest that the nature of the visual stimulus modulates its influence on tactile performance. In real-life settings, we have a wealth of experience viewing touch on a body and perceiving tactile sensations that arise because of it. Due to such repeated co-occurring experiences, there may be intersensory neural connections between visual and somatosensory systems such that visual input of touch itself may boost tactile perceptual signals leading to enhanced perception of the tactile stimulus. In addition to this, our prior experiences of viewing touch on our body and subsequently feeling touch may generate predictions about the tactile stimulus based on our stored knowledge of the visual touch stimulus. There is some evidence based on findings in other sensory modalities suggesting that content-specific expectations or predictions of a sensory state formed by prior knowledge of the stimulus can enhance perception of the stimulus (Bar, 2004; Kok et al., 2012; Stein & Peelen, 2015). On the other hand, light flash stimuli do not typically co-occur with tactile stimuli, such that we may not have experiences linking light flashes with tactile sensations. Therefore, there are likely no sensory associations between visual input of light flashes and tactile processing, nor may they facilitate predictions about the tactile stimulus. Given these differences between light flashes and visual touch, it is likely that the nature of the visual stimulus can determine the type of effect it exerts on tactile performance.

In conclusion, studies that have examined the effect of viewing tactile stimuli on the body have suggested that viewing touch may improve tactile perception. However, these studies have not examined changes in decision criterion with viewing touch, which leaves open the possibility that a liberal shift in criterion could drive improved tactile performance rather than enhanced perception. To address this issue, we developed a modified version of the SSDT, using visual touch stimuli instead of light flashes, to examine the effect of visual touch on tactile sensitivity and decision criterion. If visual touch indeed enhances tactile perception as suggested in the visual touch literature, then visual touch stimuli should lead to increased tactile sensitivity (higher d’) as compared to absence of visual touch stimuli. Given evidence that visual stimuli that are not representative of visual touch (like light flashes) can still affect tactile performance through a liberal shift in decision criterion, it is important to examine whether visual stimuli that depict touch also affect the criterion. If visual touch leads to a more liberal response bias, then visual touch stimuli should lead to a reduced criterion (smaller c = more liberal bias) as compared to the absence of visual touch.

In addition to this, given our argument about how the nature of the visual stimulus could modulate its influence on tactile processing, we expected that a representative visual touch stimulus (e.g., a finger touching the body) would increase tactile sensitivity more than a non-representative visual touch stimulus (e.g., an inanimate stimulus that touches the body). For the latter, we chose to use a moving red dot since it was not representative of touch but still mimicked the motion and trajectory of the finger touching the body. Across three experiments we found that the human touch stimulus did not consistently enhance tactile sensitivity as compared to a stimulus with no visual touch, or the red dot touch stimulus. However, in all experiments, the human touch stimulus led to a consistent liberal shift in decision criterion relative to the stimulus with no visual touch, suggesting that the higher detection rates with viewed touch versus control conditions observed in the prior literature may not necessarily reflect perceptual enhancement.

## Experiment 1

In Experiment 1, participants viewed videos of a right hand, in a posture similar to their own right hand, that was either touched by a finger (human touch condition), a moving red dot (red dot touch condition), or not touched at all (static hand condition). On tactile stimulus-present trials, a vibrotactile stimulus was delivered via a stimulator to the participant’s right index finger. Participants performed a signal detection task where they had to indicate the presence or absence of the tactile stimulus on their finger on every trial, irrespective of the visual stimulus on screen.

### Methods

#### Participants

We conducted an a priori power analysis using GPower 3 (Faul et al., 2007) to determine the required sample size for a main effect of visual touch on tactile sensitivity. Given that we were interested in whether viewed touch noticeably influences perceptual processing, we designed the study to detect an effect size that would be detectable given a reasonable number of participants ("large" effect size, η^2^ = 0.15). Based on the results of the power analysis for a repeated-measures, within-factors F test (effect size, η^2^ = 0.15 [or *f* = 0.42], alpha = 0.05, power = 0.80), the required sample size for our study was 37 participants. We collected data from 46 English-speaking undergraduate students at the University of Delaware. In current and subsequent experiments, our criteria for participant exclusion, consent process and reward for participation remained the same. Participants were excluded if (a) technical difficulties arose during data collection, such as problems with the software or equipment, (b) participants did not follow task instructions, for example, failure to keep away personal devices, falling asleep during the experiment, (c) making an incorrect response on 30% or more of practice trials, (d) our model showed a poor fit to their data (described in the data analysis section), or (e) they were likely mirror-touch synesthetes, as assessed during debriefing. There were no restrictions based on participant race or ethnicity. Based on these criteria, data from four participants were excluded for technical difficulties (problems with the white noise headphones or tactile stimulators), two for failure to follow task instructions (one participant fell asleep during the experiment, and the other closed their eyes for extended periods of time). One participant was excluded for poor model-fit to their data (total = 7). This left us with 39 participants for analysis (30 females, *M* = 18.97 years, *SD* = 1.31). This final sample size met the requirements for the projected sample size of 37 participants determined by the power analysis. The University of Delaware Institutional Review Board approved the study. We obtained written informed consent from all participants before starting the study. We granted them course credit in exchange for their participation.

#### Materials

For visual stimuli, a Logitech Brio webcam was used to record experiment videos at 30 fps with a resolution of 1,920 × 1,080. The videos consisted of a right-hand (Caucasian) placed palm-down on a wooden table, with a stimulator attached to the dorsal surface of the index fingertip. The hand occupied approximate center position on the screen and the fingertips reached the midline of the screen center. The videos were recorded from a first-person perspective with respect to the observer. In the human touch condition, at the onset of the video, the right index finger of another person’s hand (also Caucasian) approached the outstretched hand from the top left corner of the screen (500 ms), touched the index fingertip of the outstretched hand (333 ms), and then retracted into the top left corner of the screen (500 ms) for a total of 1,333 ms per video. In the red dot touch condition, a filled, red circle (approximately half an inch in diameter – about the same size as the fingernail of the outstretched hand on the screen) mimicked the motion and trajectory of the finger in the human touch condition, with each action (approach, touch, and retraction) lasting as long as corresponding events in the human touch videos. In the static hand condition, the outstretched hand remained on the screen for the duration of the video (1,333 ms), but nothing else was presented on the screen in this duration.

For tactile stimuli, ten sine waves (333 ms, 200 Hz) ranging in intensity from -36 dB (weakest) to -9 dB (strongest) in intervals of 3 dB were generated on Audacity (version 2.3.0) for the experiment. This intensity range was chosen to reflect the continuum from subthreshold to suprathreshold intensities during pilot testing. These sound files were played through a computer set at 15% of its maximum volume and were amplified (PYLE PTA2 40-W stereo power amplifier) at 50% of its full amplification range. Tactile stimuli were delivered through bone conductors (BC-10 general purpose, Ortofon Microtech) connected to the amplifier.

#### Task procedures

Participants sat approximately 60 cm from a flat screen LCD monitor (16 in., ViewSonic (model number: VS13777), 60 Hz, 1,920 × 1,080 resolution) with their body midline aligned with the screen center. The experiment was run on E-Prime Version 3.0 (Psychology Software Tools Inc., Pittsburgh, PA, USA) on a Windows 10 PC. Participants’ right hand was placed palm-down on a table in front of them to match the posture of the outstretched hand in the video. Therefore, the video hand and the participant’s own hand were matched posturally and positionally but not spatially, i.e., the video hand was seen on the monitor in front of them, while their own hand was on the table. The participant’s hand was not occluded from view. Their left hand was placed over two response keys (F and J) on a keyboard, also placed on the table. A single tactile stimulator was fixed to the dorsal surface of the participant’s right index fingertip with a Velcro strap. Participants listened to white noise through headphones to attenuate any potential sounds from the tactile stimulator.

**Fig. 1:**
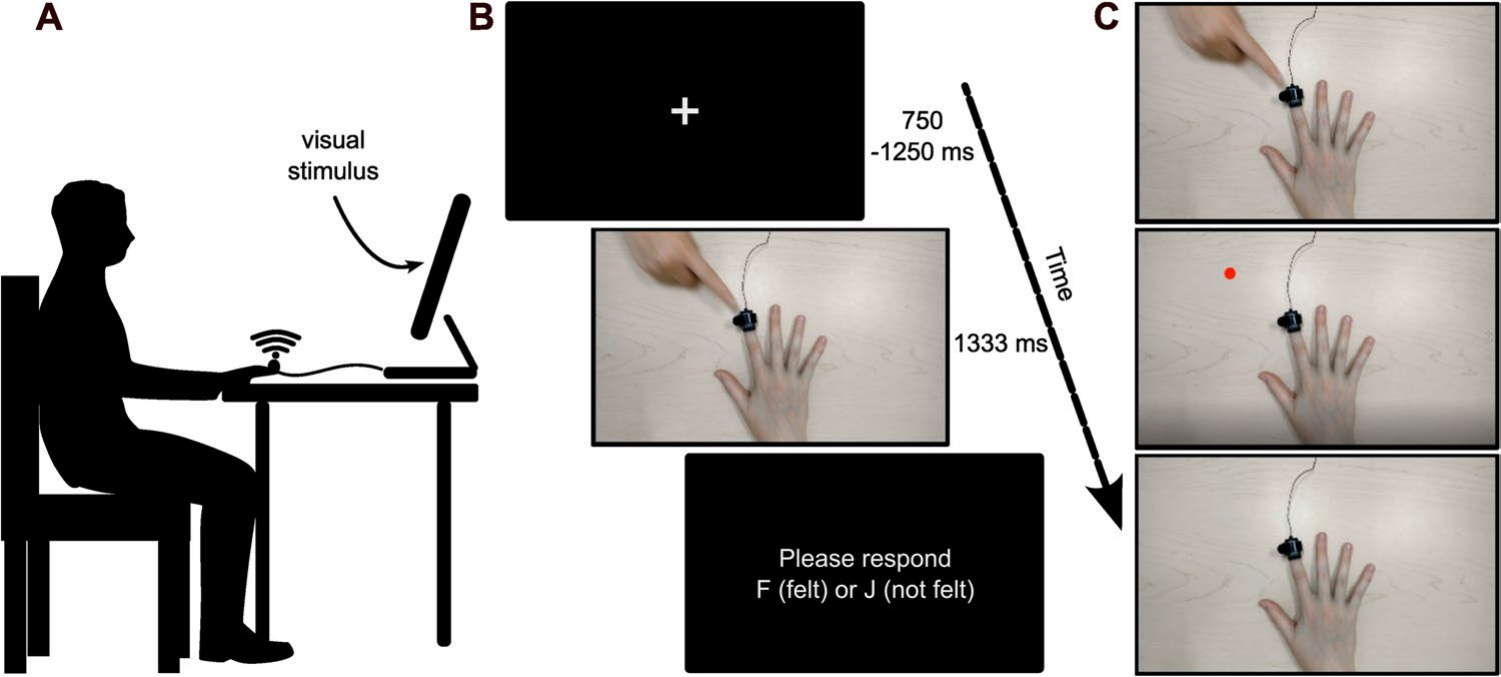
Set up and design of Experiment 1. Participants sat approximately 60 cm from the monitor with their right hand on the table and their left hand over the F and J keys on the keyboard (**A**). A tactile stimulator was strapped onto the right index finger of the participant where the tactile stimulus, if present, was delivered. Representation of the trial sequence is shown in (**B**). Each trial started with a fixation cross (750–1,250 ms), followed by one of the three videos (in this picture, the human touch condition) for 1,333 ms. A tactile stimulus, if present, occurred during this time. If the participant did not respond during the video presentation window, an alert prompted them to respond. The three types of videos (**C**) that could be presented were human touch (top), red dot touch (middle) or static hand condition (bottom)

A white fixation cross cued the start of each trial; it varied in duration between 750 and 1,250 ms on every trial and was set against a black background. Each trial in the experiment consisted of one of the three videos (human touch, red dot touch, or static hand). On tactile stimulus-present trials, a vibrotactile stimulus at one of the ten intensities was delivered through the stimulator. Therefore, we adopted a 3 (type of visual stimulus) × 2 (presence/absence of tactile stimulus) factorial design in the current experiment (Fig. 1). The onset of the tactile stimulus (if present) coincided with the visual touch on screen. For the static hand video where no visual event occurred, the tactile stimulus (if present) occurred in the same time frame as in the other videos. The participant was instructed to attend to tactile stimuli on their right index finger while also attending to the videos. They were asked to indicate if they did or did not feel a tactile stimulus on their own finger on every trial by keying in the relevant response with their left hand (F for tactile stimulus present and J for tactile stimulus absent) on the keyboard. The response keys and their correspondence to the tactile stimulus were held constant across all participants. If they did not respond during the video, an alert on the screen prompted them to respond after which it proceeded to the next trial.

Participants first performed a practice block consisting of ten trials, of which the signal trials consisted only of suprathreshold tactile stimuli (-9 dB) to ensure that they could be detected. Participants received feedback on each trial of the practice block to ensure comprehension of the task. The practice block was followed by the main experiment in which no feedback was provided. The experiment consisted of five blocks of 114 trials. Each block contained 90 tactile stimulus trials (30 trials for human touch videos, 30 for red dot touch videos, and 30 for static hand videos) and 24 no-tactile stimulus trials (eight trials for each type of video) presented in random order. The 30 tactile trials per video type consisted of three trials at each tactile intensity (10 intensities × 3 trials per condition, per block). The experiment lasted approximately 30 minutes, including the practice session. All participants were debriefed on the purpose of the study at the end of the main experiment.

#### Data analysis

Data were analyzed using MATLAB version 9.11 (R2021b) (The MathWorks, Inc., Natick, MA, USA), JASP (JASP Team (2021). JASP (Version 0.16.0)[Computer software]), R version 4.0.2 (R Core Team, 2020), and the package *ggplot2*, version 3.3.5 (Wickham et al., 2016).

For participant exclusion, a four-parameter cumulative Gaussian function (Wichmann & Hill, 2001) was fitted to subject-level data for each condition (guess rate limits range = 0.01 to 0.5, lapse rate limits range = 0.01 to 0.1) to relate individual participants’ performance to changes in tactile intensity (adapting the code available at https://github.com/garethjns/PsychometricCurveFitting). Goodness-of-fit of the function to the data was given by coefficient of determination or R^2^. The R^2^ served as an indicator of how well the observed data matched the expected pattern of performance, given the varying intensities. To retain only those participants whose data showed a strong fit to the model, we set a threshold value of R^2^ 0.85 a priori such that failure to meet the cutoff in even one condition resulted in exclusion of the participant from further analysis, similar to other papers in visual attentional literature that have also excluded participants based on goodness of fit indices (Schneider & Malik, 2021; Valsecchi et al., 2010). This procedure was followed for current and subsequent experiments.

In all experiments, tactile stimulus-present responses were classified as “hits” on tactile stimulus-present trials and “false alarms” on tactile stimulus-absent trials, irrespective of the visual condition. The main variables of interest were tactile sensitivity and criterion, given by signal detection measures, d’ [z (Hits)—z (False Alarms)] and c [(-z (Hits) + z (False alarms))/2] respectively (Macmillan & Creelman, 1991). d’ and c were calculated for each visual condition (human touch, red dot touch, and static hand). Extreme proportions of 0 and 1 in d’ and c were corrected using the 1/(2N) rule (Macmillan & Kaplan, 1985). Differences in hit rate, false alarm rate, d’, and c across the visual conditions were examined using one-way repeated-measures ANOVAs with the within-subject factor of visual stimulus type (human touch, red dot touch, and static hand). In addition to this, we performed psychometric analysis of the data to compare the point of subjective equality (PSE) and discrimination sensitivity (steepness of the curve) across the visual conditions using one-way repeated measures ANOVAs (further details are included in the Online Supplementary Material). Where the ANOVA was significant, pairwise comparisons for the a priori hypotheses were carried out using paired t-tests. These analyses remained the same for all experiments, unless otherwise noted.

### Results

#### Hit rate and false alarm rate

For our hit rate ANOVA, there was a significant main effect of visual condition, *F*(2,76) = 37.193, *p* < 0.001, η^2^ = 0.495. Consistent with the evidence in the visual touch literature, pairwise comparisons showed that the hit rate for tactile detection in the human touch condition (*M* = 47.7%, *SD* = 16.66) was significantly higher than in the red dot touch condition (*M* = 45.7%, SD = 17.77), *t*(38) = 3.152, *p* = 0.003, and the static hand condition (*M* = 41.9%, SD = 17.68), *t*(38) = 7.743, *p* < 0.001. The hit rate in the red dot touch condition was also significantly higher than in the static hand condition, *t*(38) = 5.850,* p* < 0.001. There was no significant effect of the visual condition on false alarm rate, *F*(2,76) = 0.456, *p* = 0.636, η^2^ = 0.012 (Table 1).

**Table 1:** Mean hit rate, false alarm rate, d’ and c values for each condition in Experiments 1 and 2

Experiment	Condition	Hit rate (%)	FA rate (%)	d’	C
Experiment 1	Static hand	**41.9** (17.7)	**2.5** (1.9)	**1.78** (0.5)	**1.10** (0.2)
	Red dot touch	**45.7** (17.8)***	**2.2** (1.3)	**1.90** (0.4)*	**1.06** (0.2)
	Human touch	**47.7** (16.7)***^##^	**2.7** (1.9)	**1.92** (0.5)*	**1.02** (0.2)**
Experiment 2	Static hand	**39.4** (18.1)	**1.3** (0.9)	**1.81** (0.4)	**1.19** (0.2)
	Red dot touch	**41.4** (18.7)**	**1.9** (1.3)	**1.81** (0.4)	**1.14** (0.2)*
	Human touch	**44.9** (17.9)***^###^	**3.2** (1.7)**^#^	**1.77** (0.4)	**1.02** (0.3)***^###^

% Values are expressed as a percentage of the total number of trials in each category (standard deviation in parentheses)

* represents differences compared to the static hand condition

# represents differences for human touch compared to the red dot touch condition

* *p* < 0.05, ** *p* < 0.01, ***p < 0.001

# *p* < 0.05, ## *p* < 0.01, ###* p* < 0.001

#### Sensitivity (d’)

**Fig. 2:**
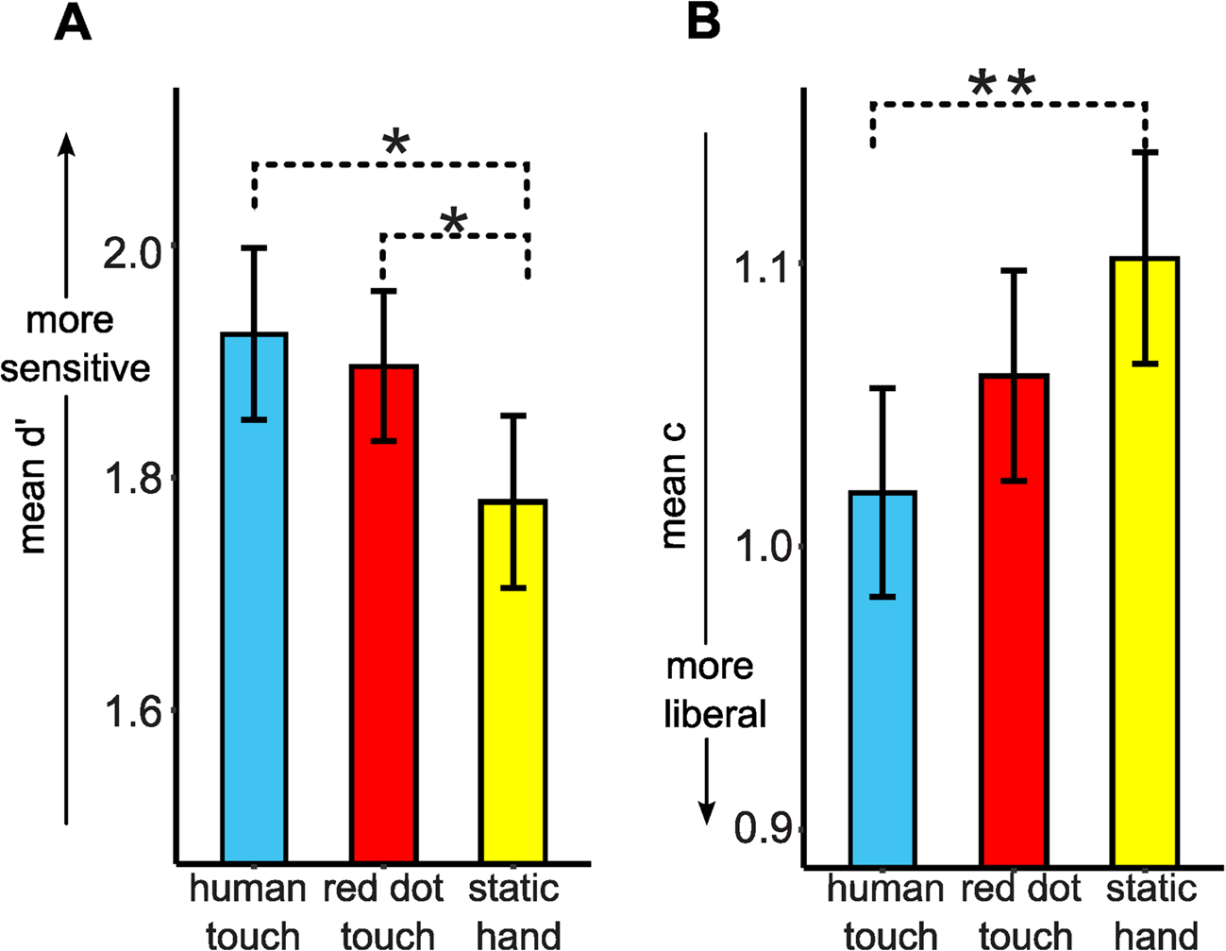
Results of the Signal Detection Analysis for Experiment 1. Results of the d’ analysis (**A**) show that tactile sensitivity (d’) was higher for human touch (HT; blue/medium gray bar) and red dot touch (RDT; red/dark gray bar) conditions compared to the static hand condition (SH; yellow/light gray bar). Results of the criterion analysis (**B**) show that c was lower (more liberal) for the human touch compared to the static hand condition. Error bars indicate standard error of the mean. * *p* < 0.05, ** *p* < 0.01

Results of the ANOVA for d’ showed a significant effect of visual condition on d’, *F*(2,76) = 4.153, *p* = 0.019, η^2^ = 0.141 (Table 1). Pairwise comparisons showed that d’ for the human touch condition (*M* = 1.92, *SD* = 0.46) was significantly higher than for the static hand condition (*M* = 1.78, *SD* = 0.46), *t*(38) = 2.502, *p* = 0.017, but not for the red dot touch condition (*M* = 1.90, *SD* = 0.40), *t*(38) = 0.497, *p* = 0.622. The d’ for the red dot touch condition was also significantly higher than for the static hand condition, *t*(38) = 2.572, *p* = 0.014 (Fig. 2A).

#### Criterion

Results of the ANOVA for c showed a significant effect of visual condition on c, *F*(2,76) = 4.455, *p* = 0.015, η^2^ = 0.105. Pairwise comparisons showed that criterion was significantly more liberal (smaller c) for the human touch condition (*M* = 1.02, SD = 0.23) than for the static hand condition (*M* = 1.10, SD = 0.23), *t*(38) = 3.199, *p* = 0.003. No other comparison was significant (static hand vs. red dot touch, *t*(38) = 1.452, *p* = 0.155; red dot touch vs. human touch, *t*(38) = 1.441, *p* = 0.158) (Fig. 2B).

### Discussion

Experiment 1 examined whether viewing touch enhances tactile sensitivity and whether it affects the decision criterion as a function of visual touch stimulus type. As hypothesized, tactile sensitivity improved when viewing a human touch stimulus as compared to the static hand condition. Tactile sensitivity also improved in the red dot touch relative to the static hand condition. Moreover, subjects were more liberal in their decision to report a tactile stimulus when viewing human touch as compared to viewing a static hand/no touch stimulus. Therefore, in addition to the observer’s sensitivity to the stimulus, viewing human touch also affected their decision processes with respect to the tactile stimulus.

In Experiment 1, we did not find evidence for enhanced performance (increased d’) when viewing a representative visual touch stimulus (human touch) versus a non-representative stimulus (red dot touch). One potential explanation for the absence of evidence for this effect could be the lack of spatial congruence between the visual and tactile stimulus in the current experimental design. In Experiment 1, the participant’s own hand is placed on the table while the viewed hand is shown on an upright monitor facing the participant. Therefore, the tactile stimulus (presented on the participant’s hand) occurs at a different spatial location than the visual touch stimulus (presented on the video hand). As per principles of multisensory integration, perception of weak signals is enhanced when multimodal stimuli are presented at the same location in space, resulting in multisensory enhancement (Bolognini et al., 2005; Meredith & Stein, 1986, 1996; Stein et al., 1988). Given this, we thought that enhancing effects for human touch versus red dot touch may only be evident when the viewed and felt touch were in the same location. In Experiment 2, we manipulated the visual stimuli to be spatially congruent with the tactile stimuli across all conditions.

## Experiment 2

The aim of Experiment 2 was to determine whether spatially congruent visual and tactile signals would provide evidence for enhanced tactile sensitivity for the human touch relative to the red dot touch condition. Given evidence that spatial congruence facilitates multisensory enhancement, if the nature of the visual stimulus influences tactile sensitivity, then d’ for the human touch condition should be significantly greater than the red dot touch condition when stimuli are spatially congruent. Like in Experiment 1, we also expected to replicate the tactile sensitivity enhancement effect for viewed touch versus the static hand condition in Experiment 2. To introduce spatial congruence between visual and tactile stimuli, we manipulated the experimental setup such that the tactile stimulus seemed to occur at the same location where participants viewed touch. Furthermore, given that the only difference in manipulation between Experiment 2 and Experiment 1 was that of spatial congruence, we also compared the results of Experiment 2 with Experiment 1 to determine whether spatial congruence led to observed differences between the two experiments.

### Methods

#### Participants

As the experimental design was the same across Experiments 1 and 2 and power was also calculated for the effect of visual touch on tactile sensitivity in Experiment 1, we based our sample size estimation for the current experiment on the results of the a priori power analysis for Experiment 1. Based on this, the required sample size was 37 participants. We collected data from 47 English-speaking undergraduate students at the University of Delaware, none of whom participated in Experiment 1. As per the participant exclusion criteria, data from seven participants were excluded for the following reasons: Two participants were excluded because the stimulator was strapped on too tightly to their finger, and one because their own hand moved such that it was not aligned with the video hand. One participant fell asleep during the experiment. and two participants’ data showed poor model fit. Finally, one participant self-reported that they were a mirror-touch synesthete. This left us with 40 participants for analysis (33 females, *M* = 19 years, *SD* = 1.06).

#### Materials and procedure

**Fig. 3:**
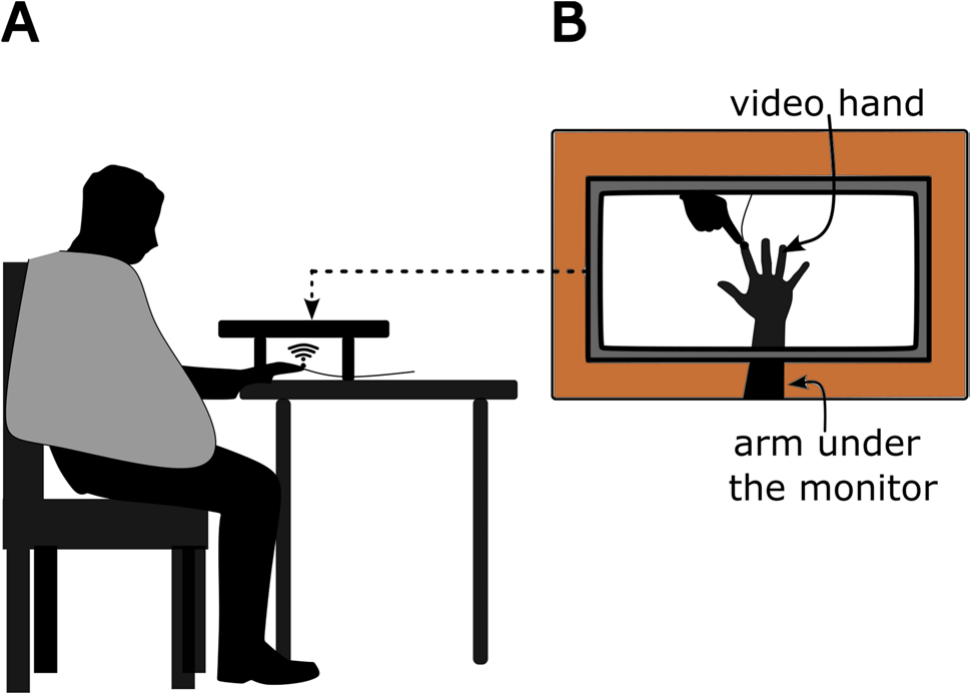
Set up for Experiment 2. Participants sat with their right hand under the presentation monitor, with their hidden hand matched in position with the viewed hand on the screen (**A**). A dark-colored cape covered the participant’s right arm from the shoulder to the wrist. The left hand was placed over the response keys on the keypad. Image of the presentation monitor is shown in (**B**). The monitor was raised using wooden blocks on either end to accommodate the participant’s hand and wrist under it. A tactile stimulator was strapped to the participant’s right index finger (under the monitor) where the tactile stimulus was delivered. The video hand was approximately the same size and at the same location as the participant’s hand, such that the touch on screen appeared to coincide with the touch felt on the hidden hand

The materials and procedure in Experiment 2 were the same as in Experiment 1, except for the following differences: In Experiment 2, the original monitor was replaced with an Acer 19.5 in. LCD monitor (1,920 × 1,080) positioned directly over the participant’s stimulated right hand (Fig. 3). The response input device was now a keypad (Sunreed Wired Numerical keypad, M/N: SK-051) instead of the keyboard. Number “4” (on the left side of the keypad) and the “ + ” key (on the right side of the keypad) were labeled as “Y” and “N” to correspond to “tactile stimulus present” and “tactile stimulus absent” responses, respectively. The videos were the same as in Experiment 1. Care was taken during setup to ensure that the video-hand occupied approximately the same amount of space on the table and adopted a similar position as the participant’s hand under the monitor, and the stimulator cable was in the same position in the videos as on the participant’s finger. Given this setup, the visual touch on the video hand appeared to coincide with the location of tactile stimulation on the participant’s finger. Furthermore, the participant’s right arm was covered with a dark cape from the shoulder to the wrist to reduce sources of discrepancy between visual and proprioceptive signals.

Given the increased visuoproprioceptive congruence between the video hand and the participant’s own hand, we expected participants to perceive a sense of embodiment for the video hand in this experiment. We hypothesized that such a setup may facilitate embodiment of the video hand at different levels across participants, leading to observed individual differences in perceived embodiment. If the level of perceived embodiment for the video hand affected sensitivity to the tactile signal or the participants’ decision criterion, one possibility is a correlation between level of perceived embodiment and participants’ d’ and/or c score. To explore the relationship between perceived embodiment and tactile sensitivity and criterion, we administered a five-item questionnaire at the end of the experiment aimed at assessing perceived embodiment. The questionnaire items were adapted from an existing embodiment scale (Botvinick & Cohen, 1998) and were as follows: (1) “The hand on the screen felt like it was my hand”; (2) “The hand on the screen felt like it was part of my body”; (3) “It felt like I was looking directly at my hand, rather than an image”; (4) “The hand on the screen felt like it was in the same location as the hand under the screen”; (5) “It felt like I could feel the touch where I saw the hand on the screen being touched.” The five items were grouped into three categories, based on the recommended classification by Gonzalez-Franco and Peck (2018). The first three items were classified under “body-ownership,” the fourth item under “location of the body,” and the fifth item under “tactile sensations.” Participants had to respond to each statement on a visual analog scale (administered on paper) by marking a point along the line that best reflected their extent of agreement. The scale was a horizontal line of length 5.5 in., with each end of the line representing positions of “strongly agree” and “strongly disagree,” respectively.

#### Data analysis

In Experiment 2, we expected spatially congruent human touch stimuli to enhance tactile sensitivity relative to the red dot touch stimulus. We also expected to replicate effects from Experiment 1, i.e., enhanced tactile sensitivity for visual touch compared to a visual stimulus with no touch. When we did not find the expected effects, we examined whether there was evidence supporting the null hypothesis of there being no effect of the visual stimulus on tactile sensitivity through a Bayesian repeated-measures ANOVA. We analyzed the data with JASP (JASP Team (2021). JASP (Version 0.16.0)[Computer software]). For hypothesis testing, we compared the null model which posited that there was no difference in d’ between the three conditions to an alternative model which proposed that d’ in the visual touch conditions was higher than in the static hand condition. We addressed this question specifically in Experiment 2 because some of our d’ results were already in line with our predictions in Experiment 1 and we hypothesized that tactile sensitivity effects for the human relative to red dot touch condition did not reach significance in Experiment 1 due to a lack of spatial congruence.

In addition to these analyses, we compared results of Experiment 2 to Experiment 1 using a 3 × 2 mixed-effects ANOVA to determine whether our manipulation of spatial congruence played a causal role in the differences between the two experiments.

Questionnaire responses were coded by measuring the distance as a percentage of line length. The average score for the first three questionnaire items represented the score for the “body ownership” category. Since there was only one question each for the “location of the body” and “tactile sensations” category, participants’ raw score on those questions represented the score for that category. To determine whether perceived embodiment for the video-hand influenced tactile sensitivity or criterion, scores for each category of the questionnaire (“body ownership,” “location of the body,” “tactile sensations”) were correlated with average d’ and c scores for each condition.

### Results

#### Hit rate and false alarm rate

Mauchly’s test indicated that the assumption of sphericity was violated for hit rate (Mauchly’s *W* = 0.841, *p* = 0.038, ε = 0.899). We adjusted degrees of freedom and p values for sphericity using the Hyunh-Feldt correction. Results of the ANOVA for hit rate showed a significant main effect of visual condition, *F*(1.799, 70.147) = 33.151, *p* < 0.001, η^2^ = 0.459). Pairwise comparisons showed that hit rate for the human touch condition (*M* = 44.9%, *SD* = 17.93) was significantly higher than the red dot touch (*M* = 41.4%, *SD* = 18.73), *t*(39) = 6.510, *p* < 0.001, and the static hand condition (*M* = 39.4%, *SD* = 18.10), *t*(39) = 7.287, *p* < 0.001. The hit rate for the red dot touch condition was also higher than the static hand condition, *t*(39) = 2.770, *p* = 0.009.

Results of the ANOVA for false alarm rate also showed an effect of visual touch, *F*(2,78) = 7.130, *p* = 0.001, η^2^ = 0.155. The false alarm rate for the human touch condition (*M* = 3.2%, *SD* = 1.68) was significantly higher than that for the red dot touch (*M* = 1.9%, *SD* = 1.26), *t*(39) = 2.515, *p* = 0.016, and the static hand condition (*M* = 1.3%, *SD* = 0.88), *t*(39) = 3.322, *p* = 0.002. The false alarm rate for the red dot touch condition was not higher than the static hand condition, *t*(39) = 1.297, *p* = 0.202.

#### Sensitivity (d’)

**Fig. 4:**
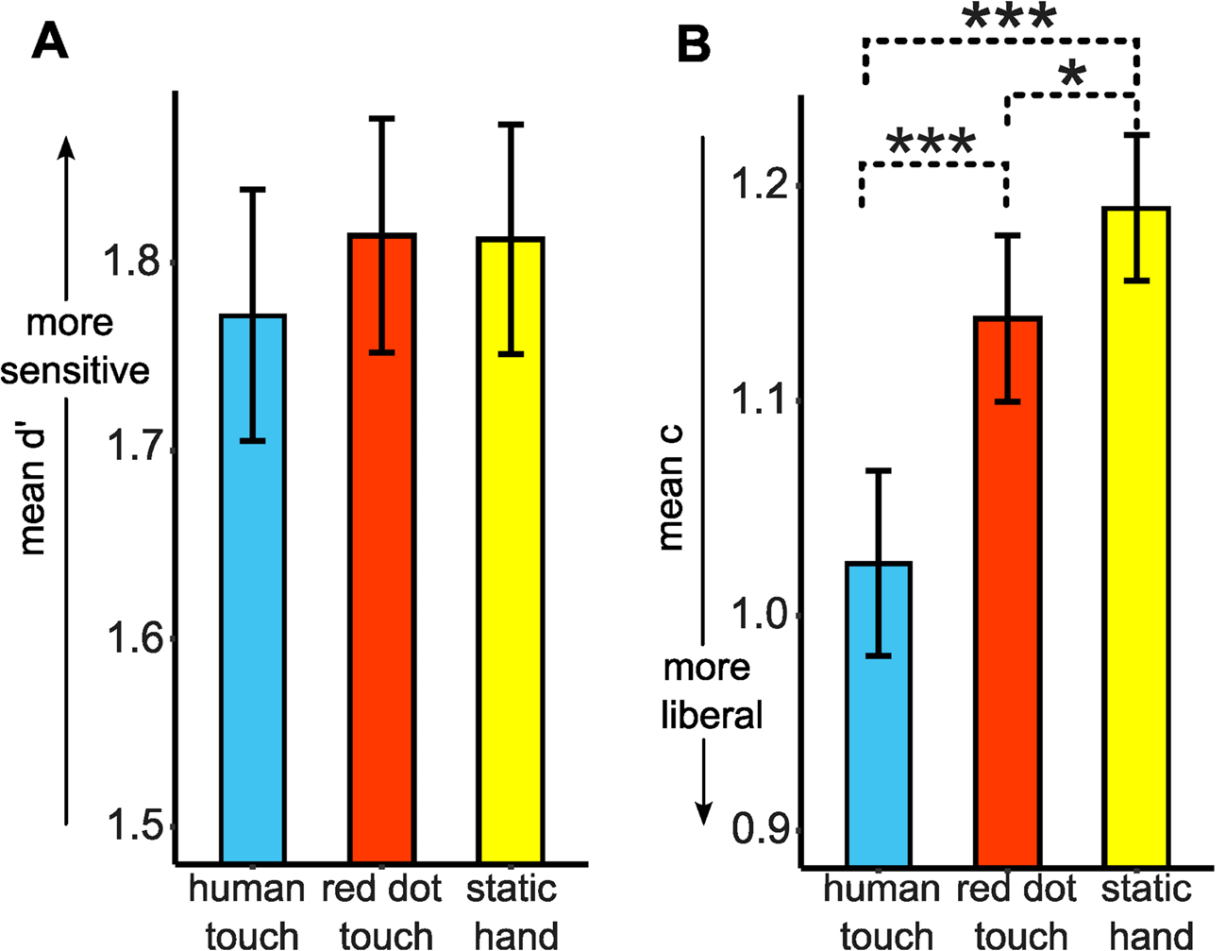
Results of the Signal Detection Analysis for Experiment 2. Results of the d’ analysis (**A**) show that tactile sensitivity did not significantly differ between the three conditions. Results of the criterion analysis (**B**) show that c was lower (more liberal) for the human touch (HT; blue/medium gray bar) compared to the static hand (SH; yellow/light gray bar) and the red dot touch (RDT; red/dark gray bar) conditions. Criterion was also more liberal for red dot touch (RDT) compared to the static hand (SH) condition * *p* < 0.05, *** *p* < 0.001

The ANOVA for d’ did not show an effect of the visual condition on tactile sensitivity, inconsistent with results of Experiment 1, *F*(2,78) = 0.480, *p* = 0.620, η^2^ = 0.012. The d’ for the human touch condition (*M* = 1.77, *SD* = 0.42) was not significantly higher than that of the static hand condition (*M* = 1.81, *SD* = 0.39), *t*(39) = 0.797, *p* = 0.430. The d’ for the red dot touch condition (*M* = 1.81, *SD* = 0.39) was also not significantly higher than that of the static hand condition, *t*(39) = -0.048, *p* = 0.962. Contrary to our hypothesis for Experiment 2, we again failed to find a significant difference between human touch and red dot touch conditions, *t*(39) = 0.790, *p* = 0.434 (Fig. 4A).

Given that we did not find evidence for our hypotheses in Experiment 2, we then asked whether the effect of visual touch on tactile sensitivity was indeed absent. If there was no effect of visual touch on d’, we should find that the observed data can be better explained by the null than the alternative model. Based on the results of the Bayesian repeated-measures ANOVA, the Bayes factor indicated that the data are best supported by a model that does not include the visual stimulus type as a predictor (BF_01_ = 8.652), indicating evidence for the null model.

#### Criterion (c)

The ANOVA for c showed a significant effect of visual condition on criterion, *F*(2,78) = 19.087, *p* < 0.001, η^2^ = 0.329. Pairwise comparisons showed a significantly more liberal criterion for the human touch condition (*M* = 1.02, *SD* = 0.27) relative to the static hand condition (*M* = 1.19, *SD* = 0.21), *t*(39) = 5.75, *p* < 0.001, consistent with the results in Experiment 1. In addition, we found that participants were more liberal in the human touch versus the red dot touch condition (*M* = 1.14, *SD* = 0.24), *t*(39) = 4.03, *p* < 0.001, and were more liberal in the red dot touch versus the static hand condition, *t*(39) = 2.06, *p* = 0.046 (Fig. 4B).

**Fig. 5:**
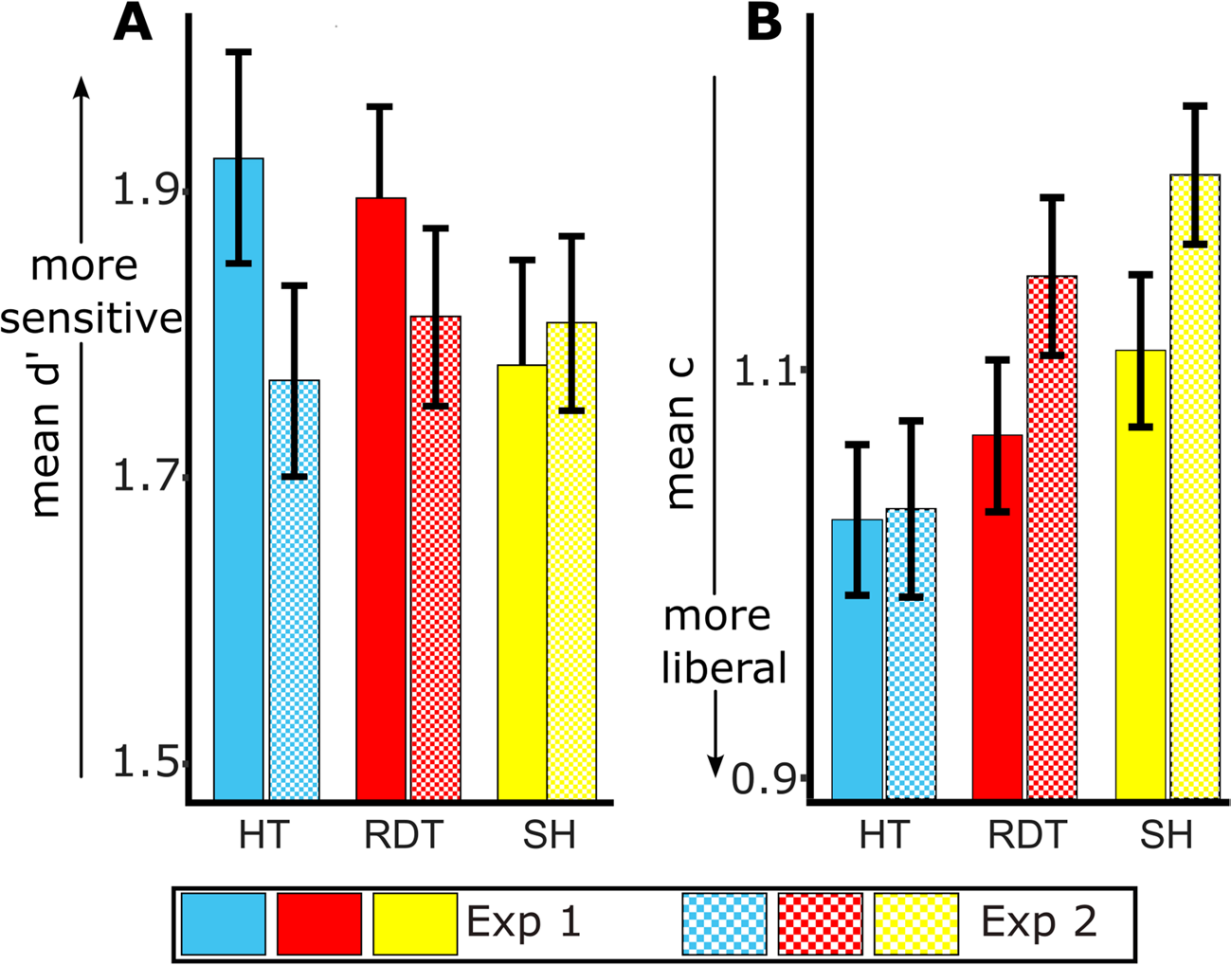
Data for Experiments 1 and 2. Mean d’ (**A**) and mean criterion (**B**) for each of the three conditions: human touch (HT; blue/medium gray bar), red dot touch (RDT; red/dark gray bar) and static hand (SH; yellow/light gray bar). Solid bars represent the data for Experiment 1 and checkered bars represent the data for Experiment 2

Since we found visual touch to enhance tactile sensitivity when visual and tactile stimuli were in different spatial locations (Experiment 1) but not when they were in the same spatial location (Experiment 2) despite no other difference between the experiments, we wanted to determine whether spatial congruence directly affected the d’ for visual touch and no touch conditions (mean d’ for Experiments 1 and 2 are shown in Fig. 5A for reference). For this, we ran a 3 × 2 mixed-effects ANOVA to examine the effect of spatial congruence and visual stimulus type on d’. For this mixed-effects ANOVA model, Mauchly’s test indicated that the assumption of sphericity was violated (Mauchly’s W = 0.923, *p* = 0.047, ε = 0.95). We adjusted the degrees of freedom and p values for sphericity using the Hyunh-Feldt correction. The results indicated a significant interaction between spatial congruence and visual stimulus type, *F*(1.9, 146.31) = 3.352, *p* = 0.040, η_p_^2^ = 0.042. There was no significant main effect of visual stimulus type, *F*(1.9, 146.31) = 1.614, *p* = 0.204, η_p_^2^ = 0.021 or spatial congruence, *F*(1,77) = 0.609, *p* = 0.437, η_p_^2^ = 0.008. Post hoc pairwise comparisons to probe the interaction effect indicated that when the video and participant hand were spatially congruent (Experiment 2), d' was greater for the static hand condition relative to the human touch condition [mean difference = -0.040, human touch < static hand]; whereas the opposite pattern was observed when there was no spatial congruence (Experiment 1; mean difference =  + 0.145, human touch > static hand], *t*(77) = 2.411, *p* = 0.018. Pairwise comparisons for the difference in d’ between red dot and static hand conditions, *t*(77) = 1.875, *p* = 0.065, and human touch and red dot touch conditions, *t*(77) = 0.907, *p* = 0.367, did not show an effect of spatial congruence.

In view of the finding that spatial congruence affects tactile sensitivity for human touch relative to the static hand condition, we also examined whether spatial congruence could affect the decision criterion for visual touch relative to the static hand condition (mean c for Experiments 1 and 2 are shown in Fig. 5B for reference). Like for d’, we ran a 3 × 2 mixed-effects ANOVA to determine if there was a significant interaction between spatial congruence and visual stimulus type on the criterion. We did not find a significant interaction effect, *F*(2, 154) = 2.676, *p* = 0.072, η_p_^2^ = 0.034. However, results indicated a main effect of visual stimulus type, *F*(2, 154) = 20.687, *p* < 0.001, η_p_^2^ = 0.212. There was no significant effect of spatial congruence, *F*(1, 77) = 1.374, *p* = 0.245, η_p_^2^ = 0.018.

Post hoc comparisons, averaged over the levels of experiment, indicated a significantly more liberal criterion for the human touch condition relative to the static hand condition (mean difference = 0.124, *p* < 0.001), for the human touch condition relative to the red dot touch condition (mean difference = 0.078, *p* < 0.001), and for the red dot touch condition relative to the static hand condition (mean difference = 0.047, *p* = 0.018) after correcting for multiple comparisons using Holm’s correction.

#### Questionnaire analysis

With the setup in Experiment 2, we also expected participants to perceive a sense of embodiment for the video hand due to the visuoproprioceptive congruence between participants’ real hand and video hand. Based on our classification of the questions, the average score for “body-ownership” questions was 52.6% while the scores for “location of the body” and “tactile sensations” questions were 81.7% and 64.9%, respectively. This suggests that perception of a shared location between real and video hand was high. We had hypothesized that, if perceived embodiment had an effect on tactile sensitivity, then there should be a significant correlation between the participant’s embodiment ratings and d’ score. Similarly, if perceived embodiment influenced decision criterion for the tactile stimulus, then we should find a significant correlation between embodiment ratings and c scores. To test these hypotheses, we correlated scores on each category of the embodiment questionnaire with d’ and c scores in each visual condition, respectively (except we did not correlate tactile sensation scores in the questionnaire with d’ or c of the static hand condition, given that there was no visual touch in this condition and therefore it was redundant to ask where they felt tactile sensations from viewed touch). To control for Type I error rates resulting from multiple comparisons, we used the Holm correction to adjust p values. After correcting for multiple comparisons, no correlation reached significance.

### Discussion

Experiment 2 was conducted to test the hypothesis that spatially congruent human touch stimuli would enhance perception of the tactile stimulus relative to the red dot touch, given that multisensory perception is enhanced when stimuli are presented at the same location in space. We also expected that spatially congruent visual touch stimuli would enhance tactile perception compared to the static hand condition, like in Experiment 1. We failed to find evidence in support of either hypothesis. Moreover, results of the Bayesian repeated-measures ANOVA favored the null hypothesis that visual touch stimuli do not influence tactile sensitivity relative to no visual touch stimuli. On comparing Experiment 1 versus 2, we also observed that tactile sensitivity for human touch relative to the static hand condition was lower with spatial congruence as compared to without spatial congruence.

Our results are surprising since we expected that spatial congruence, a feature absent in Experiment 1, would help capture differences between a representative and a non-representative visual stimulus in their effect on tactile sensitivity. Not only did we fail to observe this difference, but we also failed to replicate our original finding of tactile sensitivity enhancement with viewed touch versus static hand stimuli. Instead, our data suggest that spatial congruence seems to impair the enhancement effect for human touch versus static hand stimuli. However, in line with our findings, there is evidence in visuotactile and other modalities showing that spatial congruence does not necessarily enhance stimulus discrimination in the non-visual modality when a visual stimulus is involved. Hartcher-O’Brien et al. (2008) found that when suprathreshold visual and tactile stimuli are presented from the same location in space (therefore spatially congruent) versus different locations, participants were more likely to report only the visual stimulus versus only the tactile stimulus on bimodal trials (Colavita effect). The authors of this study hypothesized that the increased spatial congruence between the signals may lead to the two events being perceived as one, and intersensory biases favoring vision (to compensate for vision’s poor alerting capacity relative to other modalities (Posner et al., 1976)) may lead to a failure to discriminate between the two signals). Another study that applied signal detection analysis to examine the Colavita effect found that the visual dominance in bimodal auditory-visual trials was explained, at least to some extent, by a decrease in sensitivity to auditory stimuli showing that there is a negative impact on *stimulus discrimination* in the non-visual modality (Koppen et al., 2009). According to these studies, the bias towards vision may leave fewer attentional resources for processing of the stimulus in the non-visual modality, thereby leading to poorer discrimination of the stimulus in this modality (Hartcher-O’Brien et al., 2008; Koppen et al., 2009). It is possible that, in our Experiment 2, visual and tactile stimuli were perceived as a single event due to increased spatial congruence between them. Then, if attentional resources are indeed biased towards visual input, leaving fewer resources for processing of the tactile stimulus, it could explain why we failed to find an effect of visuotactile trials relative to no visual touch trials on d’, with spatial congruence versus without spatial congruence.

As for criterion effects, in addition to the liberal bias effect for human touch versus the static hand condition, we also found a liberal bias for the red dot touch versus static hand condition in Experiment 2, suggesting that participants are overall more liberal when they see a visual stimulus touch the hand versus no visual touch, under conditions of spatial congruence. Moreover, participants were also more liberal when they saw a human touch versus a red dot touch, which was not observed in the earlier experiment without spatial congruence. Furthermore, on comparing Experiment 1 versus Experiment 2, we found a main effect of visual stimulus type. Post hoc tests showed that participants were more liberal in the human touch and red dot touch relative to the static hand condition and human touch relative to the red dot touch irrespective of spatial congruence. We also found that the response criterion for human touch was not affected by spatial congruence (c = 1.02 in both experiments), which is surprising given that, in the rubber hand illusion, bringing the viewed hand closer to one’s peripersonal space increases rubber hand illusion effects (Kalckert et al., 2019; Lloyd, 2007; Samad et al., 2015; Smit et al., 2018). Our results suggest that spatial congruence may not affect decision processes for vicarious touch in the same manner that it affects the rubber hand illusion. Vicarious tactile effects are thought to occur when viewing touch on one’s own body or someone else. If so, then the effects should be the same whether the viewed touch is on one’s own body, or someone else’s body. Another potential explanation for us not seeing a change in the absolute criterion value might be because increasing spatial congruence in our experiment did not increase perceived ownership for the viewed hand which might be necessary to observe effects on the criterion. In line with this, analysis of the embodiment questionnaire responses in Experiment 2 did not indicate very high scores for “body ownership” questions (average score was 52.6%).

Despite the liberal bias observed for visual touch relative to the static hand condition in Experiments 1 and 2, it is unclear whether the liberal bias is due to the presence of touch itself or the anticipation of touch from seeing a stimulus approach the body. In the human touch or red dot touch conditions, a finger or a red dot moves towards the video hand in a predictable trajectory before touching it. This movement allows the subject to anticipate when a tactile stimulus would occur, before it actually occurs, which in turn allows them to focus their attention on the tactile stimulus window a priori. In the static hand condition, there is no visual cue which allows them to anticipate the tactile stimulus. Given this imbalance between conditions, it is possible that the liberal bias could be driven simply by anticipation for a stimulus and not by the presence of visual touch. Disambiguation between the two possibilities would require a visual stimulus that controls for the buildup of anticipation without showing touch. Previous studies have included an “approach” condition as a control for the main touch condition where the stimulus approaches the body but does not touch it (Cardini et al., 2011, 2013; Schaefer et al., 2009a, 2009b; Serino et al., 2008, 2009). Such an approach condition better controls for the effect of anticipation given that subjects cannot predict in which condition touch would occur, and therefore level of anticipation is not expected to differ across conditions. Therefore, in a subsequent experiment, we replaced the static hand condition with human hand and red dot “approach” conditions, where finger/red dot stimuli approached the video hand but did not touch it.

## Experiment 3

The aim of Experiment 3 was to determine whether the liberal bias for visual touch conditions was due to the presence of touch itself or due to anticipation of touch. To determine if the effect of visual touch on the criterion is unique from that of anticipation, we compared the criterion for visual touch conditions (that include touch and approach) with that of approach only conditions (that controls for the buildup of anticipation without showing touch). If visual touch exerted a unique influence on the criterion distinct from anticipation of the stimulus we expected to observe a simple main effect of visual touch such that criterion is more liberal for all touch versus approach conditions. The experimental setup in Experiment 3 was the same as in Experiment 2 (i.e., the visual and tactile stimuli were spatially congruent). Given that, under similar conditions, the criterion for human touch was significantly more liberal than the red dot touch condition in Experiment 2, we also expected to observe a simple main effect in Experiment 3 such that the criterion would continue to be more liberal for the human touch compared to the red dot touch condition. Given the lack of d’ effects for visual touch versus the static hand condition in Experiment 2, we did not hypothesize any d’ effects in Experiment 3.

### Methods

#### Participants

In Experiment 3, we manipulated the type of movement (touch versus approach) and visual stimulus type (human versus red dot). We conducted an a priori power analysis for a 2 × 2 ANOVA, repeated-measures design. To estimate the required sample size, we used a Monte Carlo simulation approach to calculate power given sample size, predicted means and standard deviations for each condition using the R package ANOVA power (Lakens & Caldwell, 2019). We performed 2,000 simulations for calculating power where predicted means for human touch and red dot touch conditions corresponded to mean criterion values for these conditions averaged across Experiments 1 and 2 (mean criterion for the human touch condition: 1.02 and for the red dot touch condition: 1.10). We chose mean values for the approach conditions based on predicted patterns of performance for those conditions. Since we did not expect a significant difference between human approach and red dot approach conditions, their predicted means were the same (predicted mean for the approach conditions:1.12). Our predicted standard deviation and common correlation values were based on the mean standard deviation and correlation values averaged across conditions in Experiments 1 and 2 (common standard deviation: 0.25, common correlation among within-subject factors: 0.75). We chose an alpha level of 0.05 for the tests and did not correct for multiple comparisons given that we had a priori hypotheses for the results. As per results of the simulation analysis, the required sample size to attain the desired power of 80% for the main effect of movement on criterion was 40 subjects (calculated power: ~ 83%, calculated average η_p_^2^: 0.20). We collected data from 53 English-speaking undergraduate students at the University of Delaware, none of whom participated in the previous experiments. As per the participant exclusion criteria, data from 14 participants were excluded: Two participants were excluded for technical difficulties with the stimulator and the experimental software; the experiment was discontinued for both participants. Six participants were excluded for failure to follow task instructions (poor performance on the practice block, N = 5, and failure to keep away personal devices, causing the participant to be distracted, N = 1) and six were excluded for poor model fit. This left us with 39 participants for analysis (24 females, M = 19.23 years, SD = 1.202).

#### Materials and procedure

**Fig. 6:**
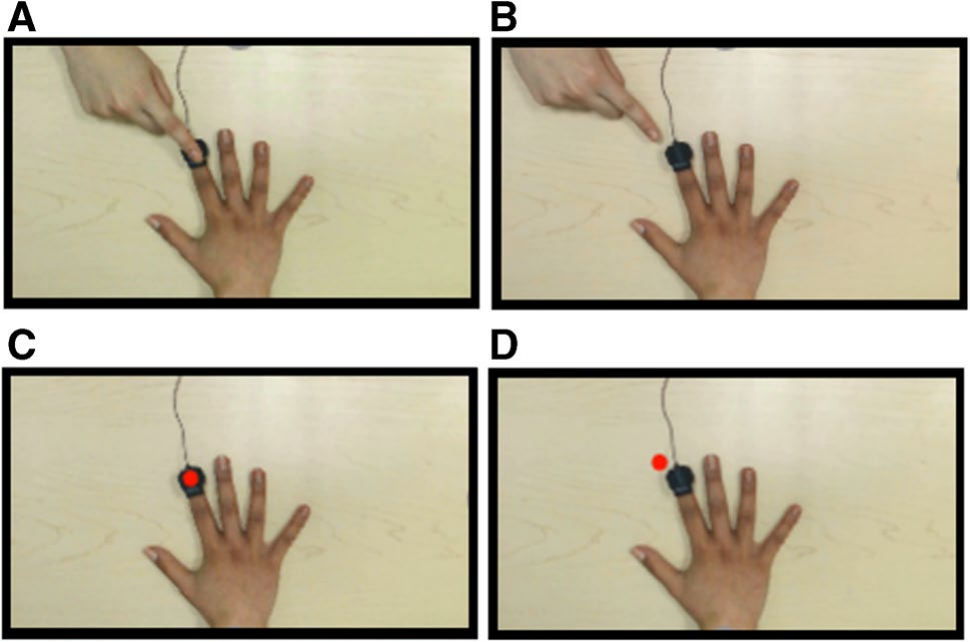
Type of videos in Experiment 3. Figures shows human touch (**A**), human approach (**B**), red dot touch (**C**), and red dot approach (**D**). In all videos, the tactile stimulus, if present, coincided with the onset of visual touch (on touch videos) or at the point at which the stimulus stops short before the video hand (on approach videos)

In Experiment 3, the setup and procedure were the same as in Experiment 2, however, the videos were altered. The two touch conditions were the same, but we replaced the static hand condition with two additional conditions – a human approach and a red dot approach condition. In both approach condition videos, the moving stimulus (finger for human touch and red dot for red dot touch) approached the video hand in the same motion and trajectory as in the corresponding touch condition videos but did not actually touch the video hand. Instead, the stimulus stopped just before touching the hand then retracted to its original position like in the touch videos (Fig. 6b, d). The timing of the approach videos matched those of the touch videos (i.e., 1,333 ms per video, 500 ms for approach, 333 ms where the position of the touching hand remained constant near the hand to be touched, 500 ms for retraction). Given that we did not find significant findings with the embodiment questionnaire in Experiment 2, we did not administer the questionnaire in Experiment 3.

To accommodate the two additional conditions, we made changes to the experimental design. The current experiment consisted of four blocks of 152 trials each. Each block contained 120 tactile stimulus trials (30 trials for human touch videos, 30 for red dot touch videos, 30 for human approach videos, and 30 for red dot approach videos) and 32 no-tactile stimulus trials (eight trials for each type of video) presented in random order. The 30 tactile stimulus trials per video type consisted of three trials at each tactile intensity (10 intensities × 3 trials per condition, per block). The experiment lasted approximately 30 min, including the practice session.

#### Data analysis

To compare differences in d’ and c between conditions, we ran a two-way repeated-measures ANOVA with the within-subject factor of visual stimulus type (human or red dot) and type of movement (touch or approach). Given our finding of liberal bias for visual touch versus static hand conditions in Experiment 2 and given previous findings in the literature showing an effect of visual touch versus approach on tactile performance, we hypothesized a main effect of type of movement on the criterion (i.e., a liberal bias for visual touch versus approach). Since we found a liberal bias for human touch versus red dot touch in Experiment 2, we also expected to find a liberal criterion effect for human touch versus red dot touch conditions in Experiment 3. Like in the previous experiment, where we failed to find an effect of the factors on d’, we conducted a Bayesian two-way repeated-measures ANOVA to evaluate evidence in favor of the null model. We also conducted a Bayesian two-way repeated-measures ANOVA to evaluate evidence in favor of the null model where we failed to find the expected effects on the criterion.

### Results

#### Hit rate and false alarm rate

Results of the ANOVA for hit rate showed a significant main effect of the visual stimulus type, *F*(1,38) = 17.138, *p* < 0.001, η_p_^2^ = 0.311. The hit rate for the human stimulus type (*M* = 44.3%, *SE* = 0.023) was greater than the red dot stimulus type (*M* = 42.3%, *SE* = 0.023). Results also showed a significant main effect of movement on hit rate, *F*(1,38) = 32.309, *p* < 0.001, η_p_^2^ = 0.460. The hit rate was higher for the touch conditions (*M* = 44.6%, *SE* = 0.022) than the approach conditions (*M* = 42.0%, *SE* = 0.023). The interaction between visual stimulus type and movement was also significant, *F*(1,38) = 8.577, *p* = 0.006, η_p_^2^ = 0.184).

On probing the interaction effect, we found a significant difference in hit rate between touch and approach for human versus red dot conditions, *t*(38) = -2.929, *p* = 0.006. The hit rate was greater for touch versus approach in the red dot condition (mean difference = 0.037) as compared to the human stimulus condition (mean difference = 0.015). Moreover, simple main effects analysis showed that hit rate for approach was significantly greater when the stimulus was a human finger (*M* = 43.6%, *SD* = 18.20) versus a red dot (*M* = 40.5%, *SD* = 17.24), *t*(38) = 5.49, *p* < 0.001.

Results of the ANOVA for false alarm rate showed no significant main effect of visual stimulus type, *F*(1,38) = 0.107, *p* = 0.745, η_p_^2^ = 0.003; no significant main effect of movement, *F*(1,38) = 0.031, *p* = 0.861, η_p_^2^ = 0.001, and no significant interaction effect between visual stimulus type and movement, *F*(1,38) = 2.990, *p* = 0.092, η_p_^2^ = 0.073 (Fig. 6).

#### Sensitivity (d’)

**Fig. 7:**
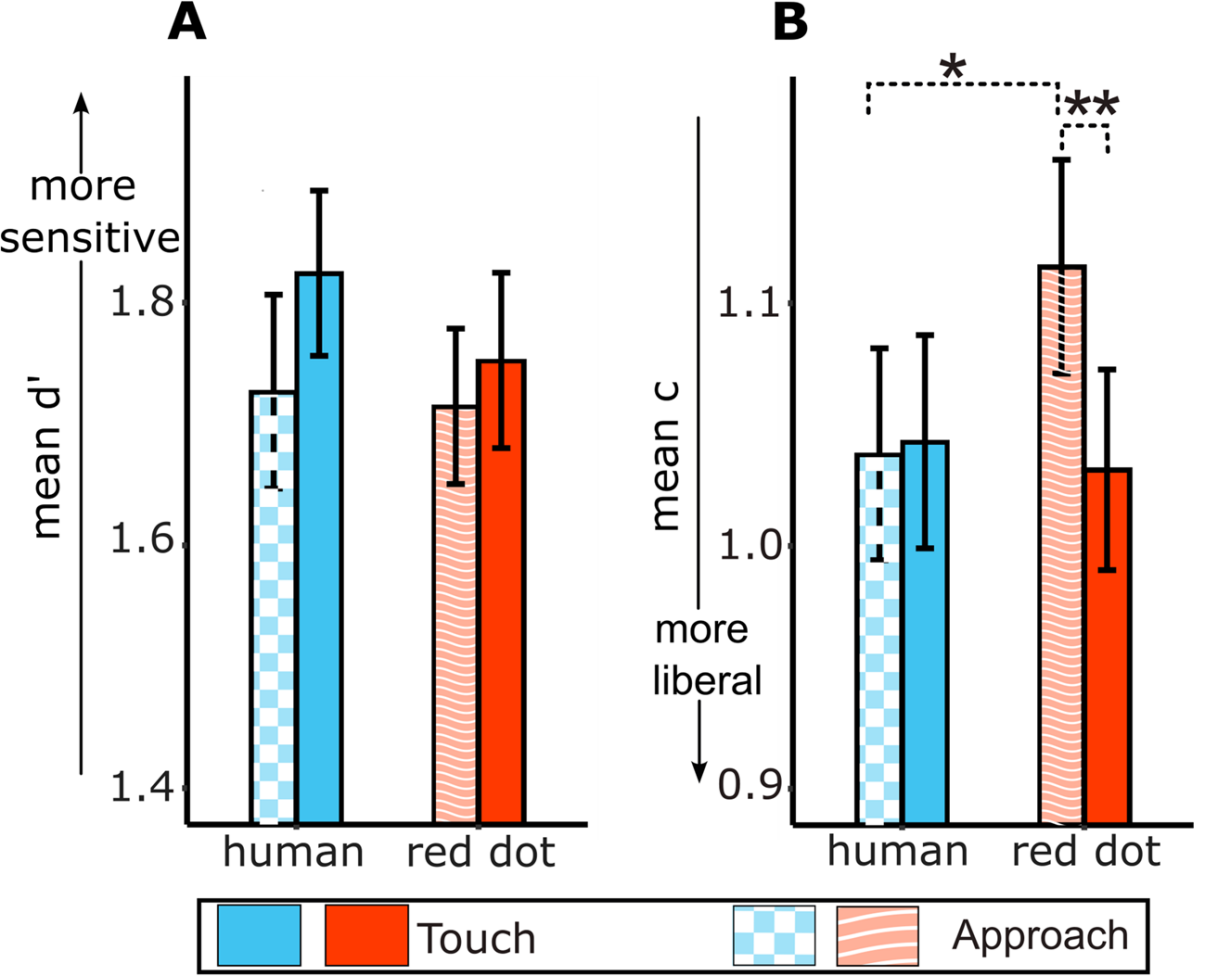
Results of the Signal Detection Analysis for Experiment 3. Results of the d’ analysis (**A**) show that tactile sensitivity did not significantly differ between the four conditions. Results of the criterion analysis (**B**) show that c was lower (more liberal) for the human approach (checkered bar) relative to the red dot approach (striped bar) condition. Criterion was also more liberal for the red dot touch (solid red/dark gray bar) compared to the red dot approach (striped bar) condition * *p* < 0.05,** *p* < 0.01

Results of the two-way ANOVA for d’ showed no significant main effect of visual stimulus type, *F*(1,38) = 1.031, *p* = 0.316, η_p_^2^ = 0.026), and no significant main effect of type of movement, *F*(1,38) = 3.714, *p* = 0.061, η_p_^2^ = 0.089, on d’. There was no significant interaction between visual stimulus type and type of movement, *F*(1,38) = 0.654, *p* = 0.424, η_p_^2^ = 0.017 (Fig. 7A).

The d’ for the human touch condition (*M* = 1.82, *SD* = 0.43) was not significantly higher than that for the human approach condition (*M* = 1.73, *SD* = 0.50), *t*(38) = 1.965, *p* = 0.057. The d’ for the red dot touch condition (*M* = 1.75, *SD* = 0.45) was also not significantly higher than for the red dot approach condition (*M* = 1.72, *SD* = 0.40), *t*(38) = 0.722, *p* = 0.474. Finally, the d’ for the human touch condition was not significantly higher than the red dot touch condition (in accordance with previous experiments), *t*(38) = 1.430, *p* = 0.161.

As with the previous experiment, we ran a Bayesian repeated-measures ANOVA to determine if the effect of visual stimulus type, movement, and/or interaction of the two factors on tactile sensitivity were null. Based on the results of the Bayesian repeated-measures ANOVA, the Bayes factor indicated that the data are best supported by a model that does not include the visual stimulus type (BF_01_ = 3.277), movement type (BF_01_ = 1.364), or interaction between visual stimulus type and movement type (BF_01_ =  ~ 3.354). However, the evidence for the null model is relatively weak given that the data are only ~ 1.4 times more likely to occur under the null model versus one that includes movement type as a factor.

#### Criterion (c)

Results of the ANOVA for criterion showed no significant main effect of visual stimulus type, *F*(1,38) = 2.330, *p* = 0.135, η_p_^2^ = 0.058, or type of movement, *F*(1,38) = 3.700, *p* = 0.062, η_p_^2^ = 0.089, on c. There was a significant interaction between visual stimulus type and type of movement, *F*(1,38) = 6.139, *p* = 0.018, η_p_^2^ = 0.139 (Fig. 7B).

On probing this interaction effect, we found that the criterion was more liberal for touch versus approach in the red dot condition (mean difference = -0.083) as compared to the human stimulus condition (mean difference = 0.005). Moreover, simple main effects showed that criterion was more liberal for human approach (*M* = 1.04, *SD* = 0.27) versus red dot approach (*M* = 1.12, *SD* = 0.28), *t*(38) = -2.60, *p* = 0.013. Criterion was also more liberal for red dot touch (*M* = 1.03, *SD* = 0.26) versus red dot approach (*M* = 1.12, *SD* = 0.28), *t*(38) = -2.954, *p* = 0.005.

Furthermore, our expected comparisons were not significant: Criterion for human touch (*M* = 1.04, *SD* = 0.27) was not more liberal compared to human approach (*M* = 1.04, *SD* = 0.27), *t*(38) = 0.198, *p* = 0.844, nor was the criterion for human touch more liberal relative to red dot touch (*M* = 1.03, *SD* = 0.26), *t*(38) = 0.442, *p* = 0.661.

Given that we did not find a significant effect of movement on the criterion as hypothesized, we ran a Bayesian repeated-measures ANOVA to determine if the effect of movement on the criterion were null. Based on the results of the Bayesian repeated-measures ANOVA, the Bayes factor indicated that the data are still best supported by a model that does not include movement type (BF_01_ = 1.211), However, similar to the result for d’, the evidence for the null model is relatively weak given that the data are only ~ 1.2 times more likely to occur under the null model versus one that includes movement type as a factor.

In our previous experiments, we found a liberal criterion for human touch relative to the static hand condition. Given that we did not find a unique effect of human touch relative to approach, we wanted to determine whether simply seeing a human-related stimulus approach the body can liberally bias the criterion relative to the static hand condition (which does not involve touch or approach). To determine whether this is the case, we compared the criterion for the human approach condition in Experiment 3 to the criterion for the static hand condition in Experiment 2 (since there was no static hand condition in Experiment 3) using an independent-samples t-test. If seeing an approaching human stimulus itself liberally biases the criterion compared to no visual touch/approach, then we should find the criterion for the human approach condition to be more liberal than that of the static hand condition. Results showed that participants were more liberal for the human approach (*M* = 1.04, *SD* = 0.27) versus the static hand condition (*M* = 1.19, *SD* = 0.21), *t*(77) = 2.759, *p* = 0.007, suggesting that even viewing a human stimulus approach a body is sufficient to liberally bias the observer. We also compared the criterion for red dot approach (*M* = 1.12, SD = 0.28) in Experiment 3 to the criterion for static hand condition (*M* = 1.19, *SD* = 0.21) in Experiment 2 using an independent-samples t-test. Results showed no significant difference in the criterion between the two conditions, *t*(77) = 1.349, *p* = 0.181.

Despite these findings, there are limitations in its interpretation given the differences in design between Experiments 2 and 3. Even though there were conditions that overlapped in Experiments 2 and 3 (human touch and red dot touch), Experiment 3 had additional, novel conditions relative to Experiment 2, which in itself might have influenced participants’ performance. For instance, when a visual stimulus appeared on the screen in Experiment 3, there could have been greater uncertainty than in Experiment 2 about whether it will touch the video hand given that the visual stimulus follows a similar trajectory for touch and approach. As evidence for this hypothesis, we found different results for comparisons between the same conditions in Experiments 2 and 3: In Experiment 2, we found a more liberal criterion for human touch relative to red dot touch (*p* < 0.001). In Experiment 3, the same comparison was not significant even though there was no difference in manipulation between the experiments with respect to these two conditions (human touch versus the red dot touch condition: mean difference in Experiment 2 [low uncertainty] = -0.11; Experiment 3 [high uncertainty] =  + 0.01). This result raises the possibility that differences in subjective confidence between the two experiments could have influenced the results and therefore comparisons between Experiments 2 and 3 should be interpreted with caution.

### Discussion

In Experiment 3, we replaced the static hand condition with approach conditions to better control for the effect of anticipation on the subject’s criterion. By doing so, we aimed to isolate the effect of visual “touch” from anticipation of the stimulus (which was preserved in the approach condition). We did not find a significant difference in criterion between human touch and human approach conditions and therefore did not have enough evidence that the effect of human touch on the criterion was unique relative to its approach. In addition, we also did not find a greater liberal bias for human touch versus red dot touch in the current experiment. On comparing Experiment 2 to Experiment 3, we found that the criterion for the human approach condition in Experiment 3 was significantly more liberal than that of the static hand condition in Experiment 2. One potential explanation for why we see a liberal bias for the human approach versus the static hand condition is that the human approach stimulus, on account of being a stimulus that has an abrupt onset on the screen, captures exogenous spatial attention. Some prior research examining the effects of exogenously captured spatial attention on perceptual performance have found such cues to liberally bias the observer (Müller & Findlay, 1987; Prinzmetal et al., 2008; Schneider & Komlos, 2008). No such cue is present in the static hand condition. If the liberal criterion effect for human approach versus static hand condition was due to exogenous spatial-attentional capture from seeing a visual stimulus approach the body, then the criterion for red dot approach should also have been more liberal compared to the static hand condition, given that the approaching red dot also captures attention exogenously like the human approach stimulus. However, we found no significant difference. Moreover, we found a significant difference between human approach and red dot approach in Experiment 3. These findings suggest that viewing a body-related stimulus approach the body, even without touching it, might liberally bias the observer’s criterion relative to viewing a stimulus with no touch/approach. One likely explanation, as we discussed in the *Introduction*, is that seeing a finger approach the body is a widely experienced phenomenon that typically results in the experience of touch. Given the high statistical frequency with which a visual stimulus of an approaching hand/finger co-occurs with the experience of tactile stimulation, it is likely that simply viewing a finger approaching the body, even without touching it, may trigger a strong expectation for a tactile stimulus. Such strong expectations may lower the criterion to report a tactile stimulus, leading to a liberal bias. This mechanism is discussed in greater detail in the *General discussion* section of the paper.

Overall, our findings do not provide enough evidence for a unique effect of touch on the criterion for human stimuli. However, they suggest that simply observing a human stimulus approach the body, without touching it, may liberally bias the observer relative to not seeing any visual stimulus. We also do not have enough evidence to state that human stimuli are unique in their liberal bias effect relative to non-human stimuli given the lack of consistent criterion effect for human touch versus the red dot touch condition.

## General discussion

Prior research on the effect of vision on tactile processing has shown increased tactile detection accuracy with viewed touch versus a visual control stimulus. The increase in accuracy could be due to two potential causes: (1) viewing touch could enhance the tactile perceptual signal, leading to higher detection accuracy, or (2) viewing touch could affect the criterion for reporting a tactile stimulus, making observers more liberal in their response when visual touch is present versus absent. To disambiguate between the two explanations, we examined the effect of viewing touch versus viewing a red dot or viewing no touch/approach stimulus (static hand) on the observer’s tactile sensitivity and decision criterion using a modified version of the Somatic Signal Detection Task (SSDT). We hypothesized that if viewing touch enhanced the tactile perceptual signal, it should enhance tactile sensitivity for an actual tactile stimulus relative to the static hand condition. On the other hand, if viewing touch made them more liberal in their response, it should lead to a liberal shift in the criterion compared to the static hand condition.

Replicating past findings in the literature, we found that viewing touch, whether from a human or a red dot, improved tactile detection accuracy as compared to the static hand condition, using percentage correct as a metric of performance. However, when we examined this effect using signal detection measures, we found that viewing human touch as compared to the static hand condition consistently led to a liberal shift in the observer’s criterion. The effect of visual touch on tactile sensitivity was only observed when visual and tactile stimuli were at separate locations in space but not otherwise. Viewing a red dot touch neither consistently improved tactile sensitivity nor did it affect the criterion. Overall, our findings suggest that viewing a human touch stimulus improves tactile detection accuracy by liberally shifting the observer’s criterion to report touch.

Evidence for improved detection rates due to changes in decision criterion have also been reported in studies examining multisensory enhancement in other sensory modalities. For example, Lippert et al. (2007) examined visual contrast detection performance in the presence or absence of a concurrent sound stimulus. In their study, concurrently presented sound stimuli improved detection rates of the visual stimulus as compared to the absence of the sound. They hypothesized that if the improved detection rates were due to low-level sensory interactions between the stimuli, leading to enhancement of the visual percept, then the improved detection rates should be observed even when the sound does not provide any additional information about the visual stimulus (e.g., its timing). However, when the sound stimulus was non-informative, there was no improvement in contrast detection rate suggesting that improved behavioral performance seen in the earlier experiment was likely due to a liberal shift in the response criterion when an informative sound stimulus was present. In this and other studies that found an improvement in task performance with concurrent multimodal stimulation (Marks et al., 2003; Odgaard et al., 2003; Yarrow et al., 2008), follow-up analyses showed evidence for a liberal response bias but no effect on stimulus discrimination. These findings provide further evidence that improved performance on a task can also be realized through changes in the decision threshold suggesting that the multimodal stimulus can affect some mechanism underlying the decision process. In line with this idea, some models of detection performance with multimodal stimuli challenge the assumption that multisensory enhancement in Yes–No detection tasks is the result of multimodal integration at early sensory stages. According to such a model, information from separate modalities is processed in their respective sensory channels, detected separately, and merged only in the decision stage. In their testing of the model, detection rates for multisensory stimuli exceeded the commonly assumed benchmark for what would be considered an early sensory-level interaction. However, they showed that such benchmarks can be exceeded even when early sensory-level interactions were improbable, calling into question some of the sensory-interaction based mechanisms proposed for multisensory enhancement (Pannunzi et al., 2015).

### Factors influencing the liberal criterion effect

In the section below, we review the main findings related to the effect of visual stimulus on the criterion, namely the role of anticipation and the effect of visual stimulus type.

Across two experiments (Experiments 1 and 2), we found a consistent liberal criterion effect for the human touch versus the static hand condition. Experiment 3 was conducted to isolate the effect of touch on the criterion from that of anticipation from seeing a stimulus approach the body. We failed to find evidence that viewed “touch” in the human stimulus condition exerted a unique influence on the criterion relative to its approach. Moreover, on comparing results for Experiment 2 versus Experiment 3, we found that simply viewing a human stimulus approach the body, without touching it, led to a liberal criterion compared to the static hand condition. These findings suggest that the consistent liberal criterion effect may be driven just by anticipation for a tactile stimulus from seeing a stimulus approach the body. Furthermore, seeing a human stimulus approach the body led to a more liberal criterion than seeing a red dot stimulus approach the body. This could be because seeing a finger approach the body may increase the expectation to feel touch, given our extensive experience viewing a hand approach the body and concurrently experiencing tactile sensations. Such expectation in turn may lower the decision threshold to report a tactile stimulus. In line with this account, increased expectation to perceive a stimulus can lead to a liberal decision bias to report the stimulus compared to when expectation is low, based on evidence in other modalities such as vision and audition (Bang & Rahnev, 2017; Dijkstra et al., 2021; Hoskin et al., 2014; Sherman et al., 2015; Wyart et al., 2012; Yon et al., 2020). This explanation would also be in line with predictive coding accounts which posit that a prediction error occurs when there is a conflict between bottom-up and top-down signals and top-down signals are weighted more heavily in decision-making regarding a stimulus (Apps & Tsakiris, 2014; Den Ouden et al., 2012; Summerfield & De Lange, 2014).

Differences in perceived level of expectation could also explain the inconsistent evidence for the liberal criterion effect of human versus non-human *touch*. The human touch stimulus led to a more liberal criterion compared to the red dot touch stimulus in Experiment 2. However, we could not replicate this effect in Experiment 3 under similar experimental conditions. We hypothesize that differences in perceived certainty regarding physical contact between the visual stimulus and the video hand could explain the failure to replicate this effect. In Experiment 2, when participants see the onset of a red dot or a human finger on the screen, they are certain that the stimulus will touch the video hand. In Experiment 3, the visual stimulus could touch the video hand or merely approach it without touching it, which introduced ambiguity in the trajectory of the visual stimulus. We directly compared the difference in criterion for human touch versus red dot touch conditions between Experiments 2 and 3 and found that increased certainty also increased the liberal bias for the human touch relative to the red dot touch condition (i.e., criterion was more liberal for human versus red dot touch in Experiment 2 versus Experiment 3). With increased certainty that visual touch will occur, the expectation that a tactile stimulus will follow might also be higher, especially when seeing the human touch stimulus for the reasons discussed above. On the other hand, when there is uncertainty whether the visual stimulus will touch the video hand, the expectation that a tactile stimulus might occur could also be low. Given the evidence regarding the role of expectation in liberally biasing the criterion, differences in expectation driven by differences in perceived certainty for visual touch could likely play a role in the observed inconsistency in results. Therefore, there might not be significant difference in tactile expectation between seeing a human stimulus versus a red dot given that they could also approach the hand without touching it.

### Potential mechanisms underlying the liberal criterion effect

While our experiments were not designed to isolate a specific mechanism for the observed criterion effects, it raises the possibility that expectations regarding perceptual states can influence how decisions about the sensory environment are made. There is evidence suggesting that our perceptual system generates top-down templates to predict sensory information from incoming bottom-up signals (Apps & Tsakiris, 2014; Den Ouden et al., 2012). These predictive templates are based on an individual’s prior knowledge and experiences of the world and allow the individual to predict future sensory states (Summerfield & De Lange, 2014). Expectations are hypothesized to increase the reliability of top-down predictive signals, especially when the bottom-up signal is ambiguous, weak, or noisy (Summerfield & De Lange, 2014), which may be one way in which they influence perceptual decision-making.

Another possibility is that changes in response criterion reflect the role of mental imagery in perceptual decision-making. Since physical stimulation on the body is typically associated with the sight of touch, there could be a strong association such that the sight of touch alone automatically activates stored mental representations of touch. Consequently, viewing physical contact (or imminent physical contact) on the body may evoke imagery of the mechanical pressure and sensation of the touch stimulus. In line with this hypothesis, there is evidence in visual perceptual literature showing that mental imagery of gratings induces a liberal bias in the observer to report the presence of congruent grating stimuli in a detection task, as opposed to not imagining the gratings, or imagining orthogonal gratings. The authors hypothesize that imagery generates sensory activity that does not influence sensory gain of the visual signal. However, the source of this activity is mistakenly attributed to the stimulus in its absence, leading to an increase in false alarms (Dijkstra et al., 2021).

While our results provide evidence for a liberal detection bias after viewing touch, we can only speculate regarding where these biases are instantiated in the brain. One possibility is that perceptual decision making for our task is supported by domain-general mechanisms that are represented in prefrontal cortex activity (Heekeren et al., 2004, 2008; Noppeney et al., 2010; Philiastides et al., 2011; Rahnev et al., 2016). However, it is also possible that these decisions are instantiated in somatosensory regions. For example, Romo et al. (2002) recorded S2 neuronal activity extracellularly from four monkeys as they performed a two-alternative forced choice frequency discrimination task for consecutively presented tactile stimuli. They found that, following the presentation of the first stimulus, neuronal activity represented the frequency encoding for this stimulus. However, following the presentation of the second stimulus, S2 activity was dominated by frequency encoding of this stimulus. Rather, its response was modulated by the frequency of the first stimulus and represented the difference in frequency for the second stimulus relative to the first stimulus. This response also correlated with the monkey’s choice on that trial, suggesting that comparisons between stimuli that inform decision-making take place in S2. Such evidence suggests that perceptual decision-making, in some part, might be instantiated in S2 (see also Romo & Salinas, 2001).

### Perceptual sensitivity with viewed touch

We had hypothesized that if viewing touch enhances the perception of the tactile stimulus, then tactile sensitivity would be enhanced when viewing touch versus not viewing touch. In our results, viewing touch (human or red dot) enhanced tactile sensitivity relative to no touch/approach only when visual and tactile stimuli were presented at different spatial locations, but not otherwise. While these findings are consistent with evidence showing increased Colavita effects with spatial congruence (see *Discussion* in Experiment 2), the absence of tactile enhancement with spatially congruent visual touch stimuli in the current study remains speculative. Future research should address the mechanisms by which relative locations of visual and tactile stimuli can influence tactile sensitivity.

We found no unique effect of the human touch stimulus (human touch versus red dot touch) on tactile sensitivity in any experiment. Viewing touch also did not enhance tactile sensitivity relative to viewing approach (Experiment 3). Therefore, even though we found increased hit rates for tactile detection with viewed touch versus no touch/approach, our data do not provide conclusive evidence that increased tactile detection was due to an enhancement of the tactile signal.

How can our findings be reconciled with the body of neuroimaging evidence showing vicarious somatosensory activity with viewed touch? The current literature proposes that vicarious activity in somatosensory areas likely reflects an enhancement of the tactile perceptual signal given that the same areas are activated for perception of an actual tactile stimulus (Blakemore et al., 2005; Cardini et al., 2011; Ebisch et al., 2008; Gillmeister, 2014; Serino et al., 2008, 2009). However, the same areas could also be activated for process(es) unrelated to tactile perception. In line with this, Carlsson and colleagues (2000) found SI and SII to be activated even by anticipation of a tactile stimulus. Anticipation for the stimulus was generated by presenting a visual cue on screen indicating an upcoming tickle stimulus. On sensory stimulation trials, a tickle stimulus followed the cue while on anticipation only trials, no stimulus followed. Participants did not report imagining or pre-emptively sensing the tickle stimulus on anticipation trials. Both tactile stimulation and anticipation activated the same areas in the brain (SI (BA 1–3) and SII), showing that activation in these areas can potentially represent non-perceptual processes. The authors speculate that this activation could be driven by top-down factors in response to anticipation for the stimulus, possibly through attentional modulation. Importantly, these findings suggest that SI and SII activation could be driven by bottom-up or top-down factors and may represent a process that does not affect perceptual processing. Likewise, evidence in other modalities shows that expectation for a stimulus can generate activity in primary sensory areas (Aitken et al., 2020; Blom et al., 2020; Kok et al., 2014) that influence perceptual decision-making by influencing the process of evidence accumulation (Feuerriegel et al., 2021; Rungratsameetaweemana et al., 2018).

One limitation in the current experiments (and prior experiments in the literature) is the discrepancy in tactile qualia evoked by the visual touch stimulus and the tactile stimulus. In most experiments, the visual touch stimulus consists of a finger or an object touching the body, while the tactile stimulus is a different kind of stimulus (e.g., vibrotactile stimulation by bone conductors, touch by monofilaments, tap by solenoid tappers). A visual stimulus showing a finger touching the body, for instance, would evoke the sensation of skin-to-skin contact, which has a distinct qualia. A vibrotactile stimulus, which does not involve skin-to-skin contact, would not evoke a sensation with such a qualia. Therefore, there is incongruence in the qualia of the sensations depicted by the visual touch stimulus and that evoked by the tactile stimulus. Given this, one possibility is that visual touch does enhance tactile perceptual signals but does not boost the same perceptual signal evoked by the tactile stimulus due to the difference in qualia. Because the current experimental design does not account for this possibility, it is not known whether the lack of evidence for a tactile sensitivity effect is due to the mismatch in the evoked qualia for visual and tactile stimuli.

## Conclusion

In summary, using signal detection measures, we found that viewing human touch stimuli leads to a liberal shift in tactile detection criterion as compared to not viewing a touch stimulus. These findings reinforce the argument that although there can be improvement in tactile accuracy with viewed touch, it is not sufficient to determine whether the improvement is due to perceptual enhancement or a shift in criterion. Our study provides clear evidence that viewing human touch can liberally bias tactile judgments of the observer which is important to account for while assessing the effects of visual touch on tactile performance.

## Supplementary Information

Below is the link to the electronic supplementary material.

Supplementary file1 (DOCX 251 KB)

## Data Availability

All data and stimulus presentation material for the current study are available on the Open Science Framework at https://osf.io/rd43w/.

## Code availability

All analysis scripts for the current study are available on the Open Science Framework at https://osf.io/rd43w/.

## Declarations

### Open practices statement

All data, analysis scripts, and stimulus presentation material for the current study are available on the Open Science Framework at https://osf.io/rd43w/. The experiments were not preregistered.
