# Supplementary material for: Viewed touch influences tactile detection by altering decision criterion
Source: Atten Percept Psychophys. 2024 Nov 5;86(8):2844–65. doi: 10.3758/s13414-024-02959-7 (PMC11652404; doi:10.3758/s13414-024-02959-7)
Supplement: Supplementary file 1 — Supplementary file1 (DOCX 251 KB) [file 13414_2024_2959_MOESM1_ESM.docx]

**Supplementary section**

As described in the main section, a four-parameter cumulative Gaussian function (Wichmann & Hill, 2001) was fitted to subject-level data for each condition (guess rate limits range = 0.01 to 0.5, lapse rate limits range = 0.01 to 0.1) to relate individual participants’ performance to changes in tactile intensity. From this function, we extracted values for the Point of Subjective Equality (PSE, or the intensity which yielded chance-level detection performance) and the discrimination sensitivity which represented the difference in intensity between detection performance at 25% and 75%. The PSE relates to the criterion in signal detection analysis as it also indicates the participant’s threshold for a detection response. A higher PSE value would reflect a more stringent criterion, whereas a lower PSE value would indicate a more liberal criterion. The discrimination sensitivity represents the slope of the function and is comparable to the d’ value in signal detection analysis. A greater discrimination sensitivity value suggests that a greater increment in stimulus intensity is needed to observe a change in performance, resulting in a shallower fit and therefore poorer sensitivity. A smaller discrimination value indicates a steeper fit and better sensitivity. Therefore, the discrimination sensitivity value is inversely related to the d’ in signal detection analysis. We examined differences in PSE and discrimination sensitivity across conditions using one-way repeated measures ANOVAs with a within-subject factor of visual stimulus type (human touch, red dot touch and static hand). Where the ANOVA was significant, we carried out pairwise comparisons for the *a priori* hypotheses using paired t-tests. These analyses remained the same for all experiments, unless otherwise noted.

# Experiment 1:

## PSE

Results of the ANOVA for PSE showed a significant effect of visual condition on PSE, *F* (2,76) = 27.075, *p* < .001, η^2^ = .416 (Fig. S1A). Pairwise comparisons showed that the PSE for the human touch condition (*M* = -21.87, *SD* = 3.47) was significantly lower than for the static hand condition (*M* = -20.45, *SD* = 3.74), *t*(38) = 6.271, *p* < .001, and for the red dot touch condition (*M* = -21.41, *SD* = 3.77), *t*(38) = 2.626, *p* = .012. The PSE for the red dot touch condition was also significantly lower than for the static hand condition, *t*(38) = 5.225, *p* = < .001 (Fig. S1B). These results indicate that the 50% threshold for detection was significantly lower for the touch versus the static hand conditions and lower for the human touch versus the red dot touch condition. In comparison to the ANOVA results for the criterion which only showed a more liberal criterion for the human touch versus the static hand condition, analysis of the psychometric data showed significant differences between all conditions. These additional effects suggest that psychometric function analyses are more sensitive to changes in participant detection threshold across conditions than the signal detection analysis.

## Discrimination Sensitivity

The ANOVA for discrimination sensitivity showed a significant effect of visual condition, *F* (2,76) = 5.216, *p* = .008, η^2^ = .121. Pairwise comparisons showed that the discrimination sensitivity value for the human touch condition (*M* = 2.50, *SD* = 1.13) was significantly lower than for the static hand condition (*M* = 3.27, *SD* = 1.31), *t*(38) = 3.752, *p* < .001, but not for the red dot touch condition (*M* = 2.63, *SD* = 1.86), *t*(38) = 0.503, *p* = .618. The discrimination sensitivity value for the red dot touch condition was also significantly lower than for the static hand condition, *t*(38) = 2.190, *p* = .035 (Fig. S1C). Since a lower discrimination sensitivity value indicates a steeper slope and better sensitivity to the stimulus, these results suggest that sensitivity was better for the touch conditions than for the static hand condition. These results are also consistent with the ANOVA results for d’ given that the same comparisons were significant in that analysis.

# Experiment 2:

## PSE

Mauchly’s test indicated that the assumption of sphericity was violated for PSE (Mauchly’s *W* = .832, *p* = .031,  ε = .892). We adjusted degrees of freedom and p values for sphericity using the Hyunh-Feldt correction. Results of the ANOVA showed a significant effect of visual condition on the PSE, *F* (1.784, 69.568) = 10.310, *p* < .001, η^2^ = .209 (Fig. S2A). The PSE for the human touch condition (*M* = -20.66, *SD* = 3.63) was significantly lower than for the static hand condition (*M* = -19.70, *SD* = 3.83), *t*(39) = 4.831, *p* < .001, and for the red dot touch condition (*M* = -20.20, *SD* = 3.85), *t*(39) = 2.547, *p* = .015. The difference in PSE for the red dot touch and the static hand condition was at borderline, *t*(39) = 2.012, *p* = .051 (Fig. S2B). These results are comparable to the results of the criterion ANOVA, given that we found significant differences in criterion for comparisons across all conditions (human touch versus static hand, human touch versus red dot touch and red dot touch versus static hand).

## Discrimination Sensitivity

The ANOVA for discrimination sensitivity did not show a significant effect of visual condition, *F* (2,78) = 3.01, *p* = .055, η^2^ = .072 (Fig. S2C). These results are in line with the ANOVA results for d’ since there were no significant differences in d’ across conditions. Pairwise comparisons showed that the discrimination sensitivity value for the human touch condition (*M* = 2.54, *SD* = 1.47) was not significantly lower than for the static hand condition (*M* = 2.19, *SD* = 1.15), *t*(39) = 1.500, *p* = .142, nor for the red dot touch condition (*M* = 2.71, *SD* = 1.38), *t*(39) = 0.867, *p* = .391. However, the discrimination sensitivity value for the static hand condition was significantly lower than the red dot touch condition, *t*(39) = 2.423, *p* = .020 (Fig. S1B) indicating better discrimination in the static hand versus the red dot touch condition. This is in contrast to the d’ results for the same pairwise comparison.

# Experiment 3:

We ran a two-way repeated measures ANOVA with the within-subject factor of visual stimulus type (human or red dot) and type of movement (touch or approach) to examine their effect on the PSE and discrimination sensitivity. Where necessary, we conducted post hoc analyses using Tukey’s post hoc test.

## PSE

Results of the ANOVA for PSE showed that, unlike the results of the criterion ANOVA, there was no statistically significant interaction between the visual stimulus type and type of movement, *F* (1,38) = 2.389, *p* = .130, η^2^ = .059 (Fig. S3A). There was a significant main effect of visual stimulus type, *F* (1,38) = 20.243, *p* < .001, η^2^ = .348, and type of movement, *F* (1,38) = 9.260, *p* = .004, η^2^ = .196, on the PSE (Fig. S3B).

## Discrimination Sensitivity

Our ANOVA for discrimination sensitivity revealed a significant interaction between visual stimulus type and movement, *F*(1,38) = 5.467, *p* = .025, η2 = .126, which was not observed for our d’ analysis (Fig. S3C). Simple main effects analysis revealed that the discrimination sensitivity value for the human stimulus was lower (i.e., better sensitivity) than that for the red dot stimulus when the type of movement was approach (*p* = .007, corrected for multiple comparisons using Tukey’s method) but not touch (*p* = 1.000). There was also a significant main effect of visual stimulus type, *F*(1,38) = 5.716, *p* = .022, η^2^ = .131, but no main effect of movement, *F*(1,38) = .007, *p* = .934, η^2^ = .0001.

The significant difference in discrimination sensitivity values between human approach and red dot approach is novel compared to the absence of effect for the same comparison in the ANOVA for d’. One potential explanation for the inconsistency in results of the two analyses lies in the way the two dependent variables are computed. While both measures are reflective of the participant's sensitivity to a stimulus, they do not necessarily represent the same process. d' analysis is based on the difference in z-scores of hits and false alarms. Thus, computation of d' is sensitive to performance on both stimulus-present and stimulus-absent trials.

The discrimination sensitivity measure represents the participant's performance on stimulus-present trials as a function of stimulus intensity. This measure does not take into account performance on stimulus-absent trials. Therefore, while it is possible that participants' detection performance improves with smaller increments in stimulus intensity for one condition versus another (which would lead to a steeper slope for that condition versus the other), it does not consider how participants respond on trials where no stimulus is presented at all. On the other hand, since d' does account for this, it determines sensitivity to the stimulus in a different manner. Therefore, the two measures capture related but not identical processes.

Another possibility is that the d’ is based on subject performance (hit rate and false alarm rate) that is collapsed across all levels of stimulus intensities, including extreme values of stimulus intensities which might show lesser variance in response compared to the less-extreme values of stimulus intensities. On the other hand, computation of the discrimination sensitivity only depends on the difference in stimulus intensity between the 25^th^ and 75^th^ percent detection thresholds for each subject. Since performance at the intensities lying between these thresholds are more likely to show the most variance, it is possible that analysis of the discrimination sensitivity captures differences between conditions that are “washed out” in the analysis of d’.

**Figure 1**

**
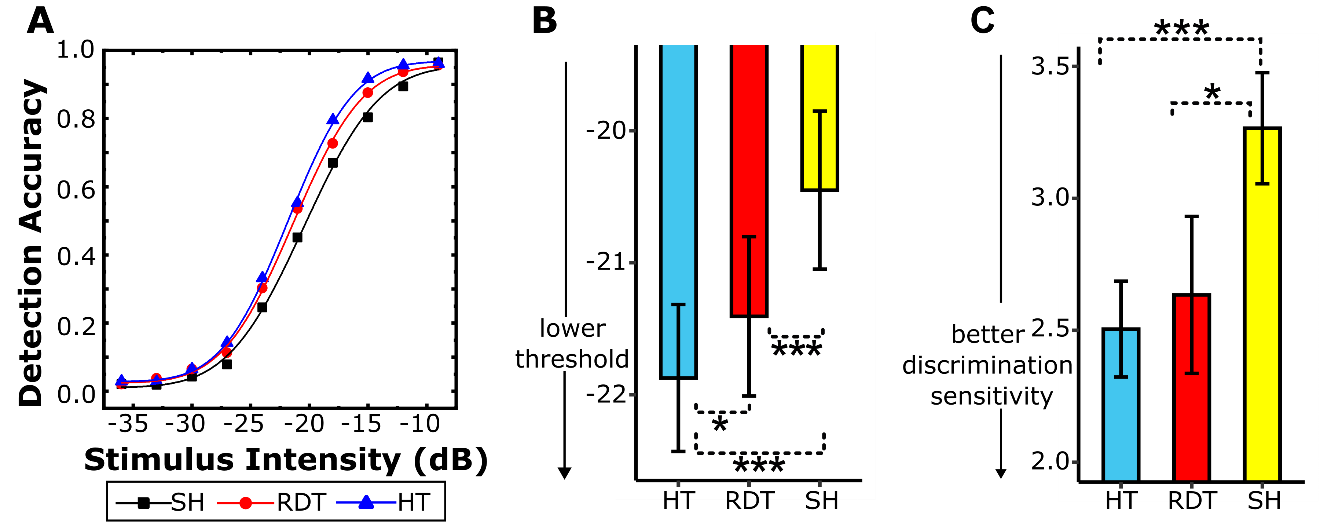
***Results of the Psychometric Analysis for Experiment 1*

*Note.* The psychometric curves for the three conditions (human touch; HT, red dot touch; RDT, and static hand, SH) are shown in **(A)**. Results of the PSE analysis **(B)** show that the PSE differed across all three conditions. PSE was lower for the human touch (blue/medium gray bar) compared to the red dot touch (red/dark gray bar) and static hand (yellow/ light gray bar) conditions. Results of the discrimination sensitivity analysis **(C)** show that discrimination sensitivity was lower (steeper slope/more sensitive) for the human touch compared to the static hand condition. Discrimination sensitivity was also lower for red dot touch compared to the static hand condition.

* *p* < .05

*** *p* < .001*.*

**Figure 2**


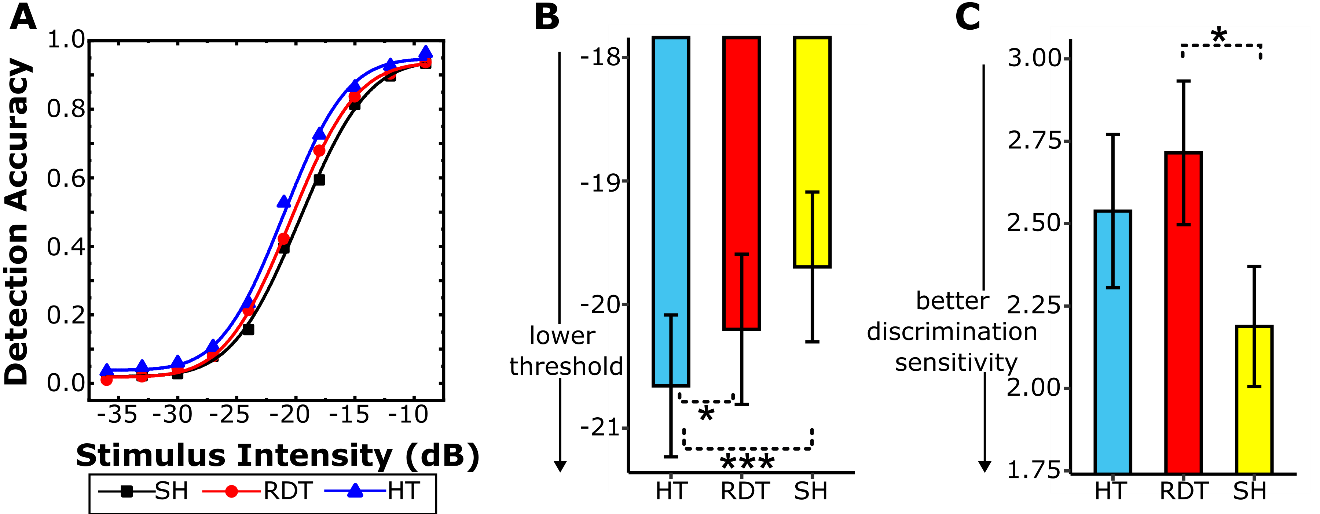
*Results of the Psychometric Analysis for Experiment 2*

*Note.* The psychometric curves for the three conditions (human touch; HT, red dot touch; RDT, and static hand; SH) are shown in **(A)**. Results of the PSE analysis **(B)** show that the PSE was lower for the human touch (blue/medium gray bar) compared to the red dot touch (red/dark gray bar) and static hand (yellow/light gray bar) conditions. Results of the discrimination sensitivity analysis **(C)** show that discrimination sensitivity was lower (steeper slope/more sensitive) for the static hand compared to the red dot touch condition.

* *p* < .05

*** *p* < .001*.*

**Figure 3**


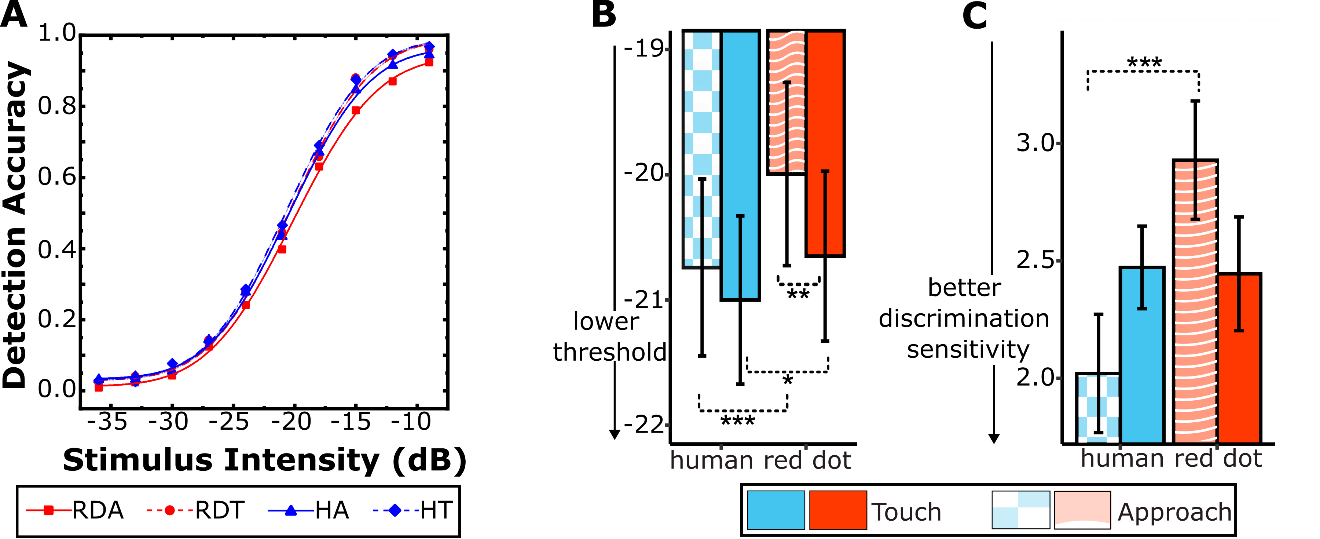
*Results of the Psychometric Analysis for Experiment 3*

*Note.* The psychometric curves for the four conditions (human touch, red dot touch, human approach, and red dot approach) are shown in **(A)**. Results of the PSE analysis **(B)** show that the PSE was lower for the human approach (checkered bar) compared to the red dot approach (striped bar) condition. PSE was also lower for the human touch (blue/medium gray bar) compared to the red dot touch (red/dark gray bar) condition, and for the red dot touch compared to the red dot approach condition. Results of the discrimination sensitivity analysis **(C)** show that discrimination sensitivity was lower (steeper slope/more sensitive) for the human approach hand (checkered bar) compared to the red dot approach (striped bar) condition.

* *p* < .05

** *p* < .01

*** *p* < .001*.*
